# Long-Term Residential Exposure to Particulate Matter and Its Components, Nitrogen Dioxide and Ozone—A Northern Sweden Cohort Study on Mortality

**DOI:** 10.3390/ijerph18168476

**Published:** 2021-08-11

**Authors:** Johan N. Sommar, Ulla A. Hvidtfeldt, Camilla Geels, Lise M. Frohn, Jørgen Brandt, Jesper H. Christensen, Ole Raaschou-Nielsen, Bertil Forsberg

**Affiliations:** 1 Section of Sustainable Health, Department of Public Health and Clinical Medicine, Umeå University, 90187 Umeå, Sweden; bertil.forsberg@umu.se; 2 Danish Cancer Society Research Center, Strandboulevarden 49, 2100 Copenhagen, Denmark; ullah@cancer.dk (U.A.H.); ole@cancer.dk (O.R.-N.); 3 Department of Environmental Science, Aarhus University, 4000 Roskilde, Denmark; cag@envs.au.dk (C.G.); lmf@envs.au.dk (L.M.F.); jbr@envs.au.dk (J.B.); jc@envs.au.dk (J.H.C.)

**Keywords:** air pollution components, carbonaceous particles, secondary inorganic aerosols, secondary organic aerosols, sea salt, mortality

## Abstract

This study aims to estimate the mortality risk associated with air pollution in a Swedish cohort with relatively low exposure. Air pollution models were used to estimate annual mean concentrations of particulate matter with aerodynamic diameter ≤ 2.5 µm (PM_2.5_), primary emitted carbonaceous particles (BC/pOC), sea salt, chemically formed particles grouped as secondary inorganic and organic aerosols (SIA and SOA) as well as ozone (O_3_) and nitrogen dioxide (NO_2_). The exposure, as a moving average was calculated based on home address for the time windows 1 year (lag 1), 1–5 years (lag 1–5) and 1–10 years (lag 1–10) preceding the death. During the study period, 1151 cases of natural mortality, 253 cases of cardiovascular disease (CVD) mortality and 113 cases of respiratory and lung cancer mortality were observed during 369,394 person-years of follow-up. Increased natural mortality was observed in association with NO_2_ (3% [95% CI −8–14%] per IQR) and PM_2.5_ (2% [95% CI −5–9%] for an IQR increase) and its components, except for SOA where a decreased risk was observed. Higher risk increases were observed for CVD mortality (e.g., 18% [95% CI 1–39%] per IQR for NO_2_). These findings at low exposure levels are relevant for future decisions concerning air quality policies.

## 1. Introduction

The association between atmospheric particles with an aerodynamic diameter less than 2.5 µm (PM_2.5_) and mortality has been well documented [1,2,3,4,5]. Nitrogen oxides (NO_x_) and NO_2_, often used as indicators of traffic emissions, have also been found to be associated with increased risk of pre-term mortality [6,7,8,9]. The main causal determinant is, however, considered to be atmospheric particles [10].

In Europe, the large European Study of Cohorts for Air Pollution Effects (ESCAPE) project, including 22 cohorts, found an association between PM_2.5_ and an increased risk of all-cause mortality [4]. There was, however, a considerable heterogeneity in relative risk estimates between cohorts. The heterogeneity may partly be due to differences in PM sources and composition [11,12].

Studies on the long-term effect on mortality by constituents of PM are scarce. Identifying constituent-specific relative risks are important for policy making aiming to mitigate air pollution associated health effects. A review by Luben et al. (2017) [13] identified long-term black carbon (BC, or elemental carbon (EC)) exposure, an element of PM, as associated with mortality due to coronary heart disease [14] and ischemic heart disease [11,15]. The review did, however, not suggest BC to be stronger associated with mortality per IQR than PM_2.5_. Another meta-analysis also found that elemental carbon was associated with all-cause mortality with a much bigger effect than PM_2.5_ per µg/m^3^ [3].

Cohort studies from the US found that long-term exposure to sulfate (SO_4_^2−^) was associated with mortality (all-cause, cardiopulmonary disease and lung cancer mortality; [11,16,17,18]). A review and meta-analysis of long-term exposure to fine particulate matter constituents and natural mortality found statistically significant increased risks associated with BC, nitrate (NO_3_), zinc (Zn) and silicon (Si) [19]. A large heterogeneity was observed for copper (Cu) and iron (Fe). The meta-analysis also found support for associations between Fe, nitrate, Zn, Si and CVD mortality, whereas there were not sufficient number of studies to conduct a meta-analysis for respiratory mortality. Cu, iron (Fe), potasium (K), nickel (Ni), sulfur (S), Si, vanadium (V) and Zn were associated with natural-cause mortality in a pooled analysis of eight European cohorts [20]. Within a recent study of the Danish Diet, Cancer and Health cohort all-cause mortality increased with NO_2_, O_3_, PM_2.5_, BC and secondary organic aerosols (SOA) [21,22]. The association with BC and SOA remained after adjustment for PM_2.5_ in two-pollutant models.

Recent studies have indicated a steeper exposure-response curve at lower PM concentrations [1]. Allowing for a non-linear association between PM_2.5_ and log all-cause mortality risk in their analyses the relative risk estimate was 25% higher at 10 μg/m^3^ compared with at 15.7 μg/m^3^ (which was the mean across all included studies). In an updated review, the combined relative risk estimate per 5 µg/m^3^ PM_2.5_ was 1.04, but 1.08 (95% CI 1.06–1.11) for the five studies with a mean concentration below 10 µg/m^3^ [23]. This risk increase was thus more than twice as high as when also including studies with higher exposure levels. The results also suggest higher risk estimates in relation to local than regional exposure. Further, a high geographical resolution of the modelling results used for the exposure assessment has been associated with higher risks [24].

This study aims to estimate the mortality risk associated with air pollution measured as PM_2.5_, primary emitted carbonaceous particles (BC and primary organic carbon (pOC)), sea salt, chemically transformed particles grouped as secondary inorganic and organic aerosols (SIA and SOA), ozone (O_3_) and nitrogen dioxide (NO_2_).

## 2. Methods and Materials

### 2.1. Study Participants

Our study period ranged from 1 January 1990 to 31 December 2014. Residential address histories were obtained for all cohort participants through linkage by using personal identification numbers to mandatory records of residential addresses at Statistics Sweden. These residential addresses were then geocoded by automatically matching against the Swedish Mapping Cadastral and Land Registration Authority Databases. When needed, addresses were manually checked and corrected for inconsistencies and assigned geographical coordinates.

The Västerbotten intervention program (VIP), including Umeå municipality, is a program where the population in the County is invited to a health examination the year they turn 40, 50 and 60 (and during some years also 30) years old [25]. The screening was initiated to identify individuals at high risk of cardiovascular disease and diabetes through interview questions and clinical measurements. The interview questions provide information about risk factors such as social situation, education, diet and physical activity. VIP was initiated in parts of the County in 1985. So far more than 100,000 individuals have participated in the program. Participation rates have ranged between 48 and 67%. Between 1995 and 2005, the participation rate was 66–67%. A dropout rate analysis in 1998 indicated only a small social selection bias. The 43,216 individuals that lived in Umeå municipality were included in this study.

### 2.2. Mortality Outcomes

Cause-specific mortality was determined by linkage of national personal identification numbers to the death registries of the Swedish National Board of Health and Welfare. We used the International Code of Diseases ICD-9 001–779 or ICD-10 A00–R99 to define deaths by natural causes, ICD-9 400–440 or ICD-10 I10–I70 to define deaths in CVD and ICD-9 460–519 or ICD-10 J00–J99 to define non-malignant respiratory mortality.

### 2.3. Exposure Assessment

The exposure assessment is based on a multiscale modelling system, whereby both the long-range transported air pollution, the contribution from local sources and the variations in meteorology are included. The system is based on a coupling between two models (DEHM and UBM) described in the following.

The Danish Eulerian Hemispheric Model (DEHM) is a 3D chemistry-transport model including a comprehensive description of 80 chemical components [26]. The model covers the Northern hemisphere and includes subdomains over Europe and Northern Europe with higher and higher spatial resolution (150 km → 50 km → 16.67 km). The model describes the overall processes related to chemical transformation, transport and removal of air pollution based on the input of anthropogenic emissions (from the European Monitoring and Evaluation Programme (EMEP) database [27]) and meteorological parameters (from the Weather Research and Forecasting (WRF) model [28]). Natural emissions of biogenic volatile organic compounds (BVOC’s) from the biosphere and sea salt from marine surfaces are estimated within the model as a function of e.g., incoming solar radiation, air temperature and wind speed [29,30] and natural emissions of e.g., NO_x_ are included from the Global Emissions InitiAtive (GEIA) database [31].

In order to include the contribution from local sources in higher detail, the regional model is coupled to the Urban Background Model (UBM), which is a Gaussian plume-in-grid model including simple photochemistry The UBM model is suitable for estimating the transport and dispersion of the main air pollutants influenced by local scale emissions [32]. UBM is in this study setup for a large domain covering the Nordic countries with a resolution of 1 km × 1 km. Local emissions are based on the new NordicWelfAir emission inventory [33] with 1 km × 1 km resolution, while hourly regional background concentrations and meteorology data are interpolated from the DEHM model results described above.

The output of the UBM model is hourly concentrations of the primary emitted gases nitrogen-dioxide (NO_2_), carbon-monoxide (CO), sulphur-dioxide (SO_2_) and ammonia (NH_3_) that are directly emitted from combustion processes and the agricultural sector, for example, and the gas ozone (O_3_), chemically formed within the atmosphere. Furthermore, the model outputs hourly concentrations for a variety of particles with a diameter less than 2.5 µm and 10 µm, and the sums of the particles that contribute to the two size classes are referred to as PM_2.5_ and PM_10_. These sums cover the primary/directly emitted particles black carbon (BC), organic carbon (pOC) and mineral dust related to power plants, traffic and wood stoves, but also the particles formed within the atmosphere. The latter is the secondary inorganic aerosols (SIA), which are the sum of sulphate (SO_4_^2−^), nitrate (NO_3_^−^), related to combustion processes and ammonium (NH_4_^+^), related to agriculture, as well as secondary organic aerosols (SOA) formed from organic precursors emitted mainly from the biosphere. Finally, the model includes sea salt aerosols. This setup secures that the primary components emitted locally are included in UBM with the high resolution corresponding to the emission inventory.

#### 2.3.1. Model Evaluation

The DEHM model is continuously evaluated against observations/other models [34,35,36] and is currently part of the Copernicus Atmosphere Monitoring Service (CAMS) and evaluated online against European observations (see https://www.regional.atmosphere.copernicus.eu/ (accessed on 5 August 2021)).

The coupled system DEHM/UBM has been applied in a number of studies of health effects related to air pollution [22,37,38,39,40] and is extensively evaluated for Denmark, e.g., in connection with the Danish monitoring programme as well as international studies [41,42]. For the current study, DEHM/UBM has been evaluated against PM_2.5_ observations from an urban background site. Overall, the model underestimates the PM_2.5_ level by ca. 30%, which is mainly due to the fact that the observations include the water content of the particles, for example, while the models only represent the dry part of the PM_2.5_. The correlation between daily and monthly mean observations and the model is between 0.6 and 0.7, indicating that the model is able to reproduce the observed seasonal and day-to-day variations in the urban background level of PM_2.5_ in Umeå.

Examples of the modelled distributions of NO_2_, O_3_ and PM_2.5_ and their components across the Umeå region are given in Supplementary Material Figure S1a–h. These illustrate the impact of the local emissions resulting in enhanced concentrations and gradients across the urban area as well as the impact from long-range transport of sea salt over the Gulf of Bothnia, for example.

#### 2.3.2. Individual Exposures

Finally, using the 1 km × 1 km grid, the resulting modelled annual mean concentrations were added to each study participant using geocodes for the home addresses. The address tracking allowed us to account for any changes in address during the study period 1990–2014. The annual mean concentrations were based on the modelled hourly time series for the full period.

### 2.4. Confounders

Associations were adjusted for potential confounding by including sex, calendar year, smoking status (current, former, never smoker), fruit and vegetable intake, body mass index (BMI), BMI^2^, alcohol consumption (daily, weekly, seldom, never), physical activity (sedentary, moderate, intermediate or vigorous), marital status (single, married or living with partner, no answer), education level (primary school or less, up to secondary school or equivalent, university degree or more, no answer), occupation status (gainfully employed, unemployed/not gainfully employed, retired, no answer). Area level confounders included mean neighbourhood individual income in persons of working age, the proportion of inhabitants (PI) between 30 and 60 years with disposable family income in the lowest quartile, PI between 30 and 60 years who were unemployed, PI between 30 and 60 years whose highest attained education was categorized as basic in UNESCOs International Standard Classification of Education, proportion of households (PH) being rented, PI with no car, PI (non-married and non-cohabiting) with a child living at the same address, PI from non-western countries, number of inhabitants/km^2^ in the specified neighbourhood, by Small Areas for Market Statistics (SAMS) provided by Statistics Sweden for the calendar year 1994.

### 2.5. Statistical Methods

Cox-proportional hazard models were used to estimate hazard ratios of mortality associated with PM, NO_2_ and O_3_. Age was used as the underlying time variable for the baseline hazard. The regression model included adjustment for calendar year, baseline information on confounders as well as area level socioeconomy for the calendar year 1994. A principal component analysis was used to limit the dimensionality of the area level socioeconomic indicators and thereby reduce standard errors. The AIC was used to select the number of principal components to include. We censured individuals at death by other causes, the end of the study period or time of permanent emigration from the study areas. Associations were assessed using three exposure windows: the previous year (lag 1), a moving average over the last 5 years (lag 1–5) and last 10 years (lag 1–10). For inclusion, annual mean concentrations were required for at least 80% of the time window. Hazard ratios and 95% confidence intervals (CI) were expressed per interquartile range (IQR) increase in exposure, as well as per 5, 1 and 10 µg/m^3^ for PM_2.5_, BC and pOC and O_3_, respectively.

Two-pollutant models were assessed with PM_2.5_ and BC and pOC adjusted for NO_2_, and PM_2.5_, NO_2_, BC and pOC adjusted for O_3_. SIA, SOA and sea salt (SS) risk estimates were also in two-pollutant models adjusted for PM_2.5_, and models with mutual adjustment between SIA and SOA.

All statistical analyses were made using the statistical software R [43].

## 3. Results

### 3.1. Participant Characteristics

The study included 42,580 individuals with a median age at recruitment of 40 years of which 52% were women (Table 1). Their average BMI was 25 kg/m^2^, 19% were current smokers and 30% former smokers, 36% did not exercise (that required training clothes), 18% consumed alcohol every week, about 5% had low intake of fruit and vegetables, 23% did not live with a partner, 30% had primary school education or less and 6% were unemployed. In the highest tertile of PM_2.5_, a somewhat larger proportion were women, current smokers, less frequently physically active during their leisure time, had a lower education level and lower proportion of gainful employed. During 369,394 years of follow-up, 1151 deaths by natural causes, 253 deaths by CVD and 113 deaths by respiratory disease were recorded.

### 3.2. Particle Concentrations

**Figure 1:**
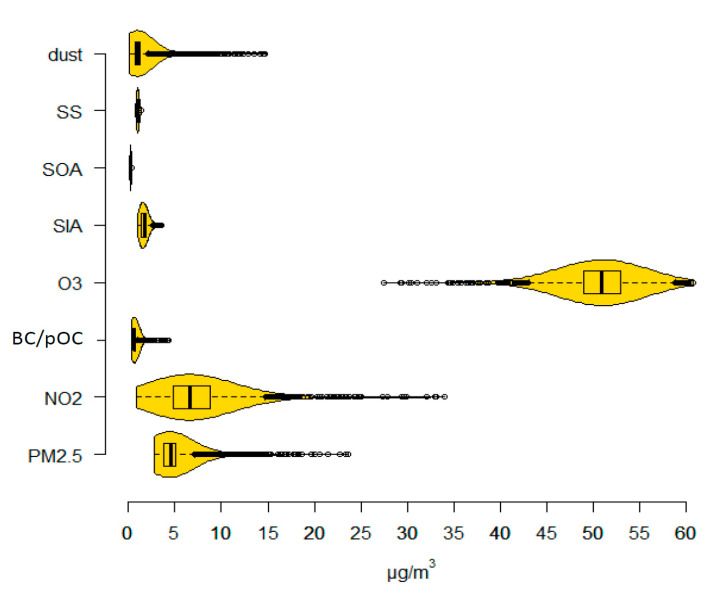
Boxplots and density functions of air pollution concentrations. SS = sea salt, SOA = Secondary Organic Aerosols, SIA = Secondary Inorganic Aerosols, O_3_ = ozone, BC/pOC = primary emitted Carbonaceous Particles, NO_2_ = nitrogen dioxide, and PM_2.5_ = particulate matter with aerodynamic diameter ≤ 2.5 µm.

Mean lag 1 concentrations of PM_2.5_, NO_2_ and O_3_ were 4.90, 7.09 and 50.76 µg/m^3^ among the person-years of follow up (Figure 1 and Table 2). For the PM_2.5_ components BC and pOC, SIA, SOA, SS and mineral dust, the means were 0.66, 1.67, 0.23 and 1.02 µg/m^3^, respectively. Lag 1–5 and 1–10 concentrations were on average higher than lag 1 concentrations due to a decreasing trend in concentrations during the study period. The coefficient of variation (CV, the standard deviation in relation to the mean) differed between calendar years (Supplementary material Table S1), but was low for O_3_, SOA and SS (on average 7–8%). Lag 1 PM_2.5_ concentrations were highly correlated with concentrations of NO_2_, BC and pOC, O_3_ and dust, whereas lower correlations were found with SIA (r = 0.47), SOA (r = 0.23) and SS (r = 0.10; Table S2a; lag 1–5 and 1–10 concentrations in Table S2b,c, respectively). The correlations between the air pollutants PM_2.5_, NO_2_, BC and pOC, O_3_ and dust were fairly stable over time, but varied for SIA, SOA and SS (data not shown). No increasing or decreasing trend in correlation was observed, however, during the study period.

### 3.3. Associations with Mortality

**Figure 2:**
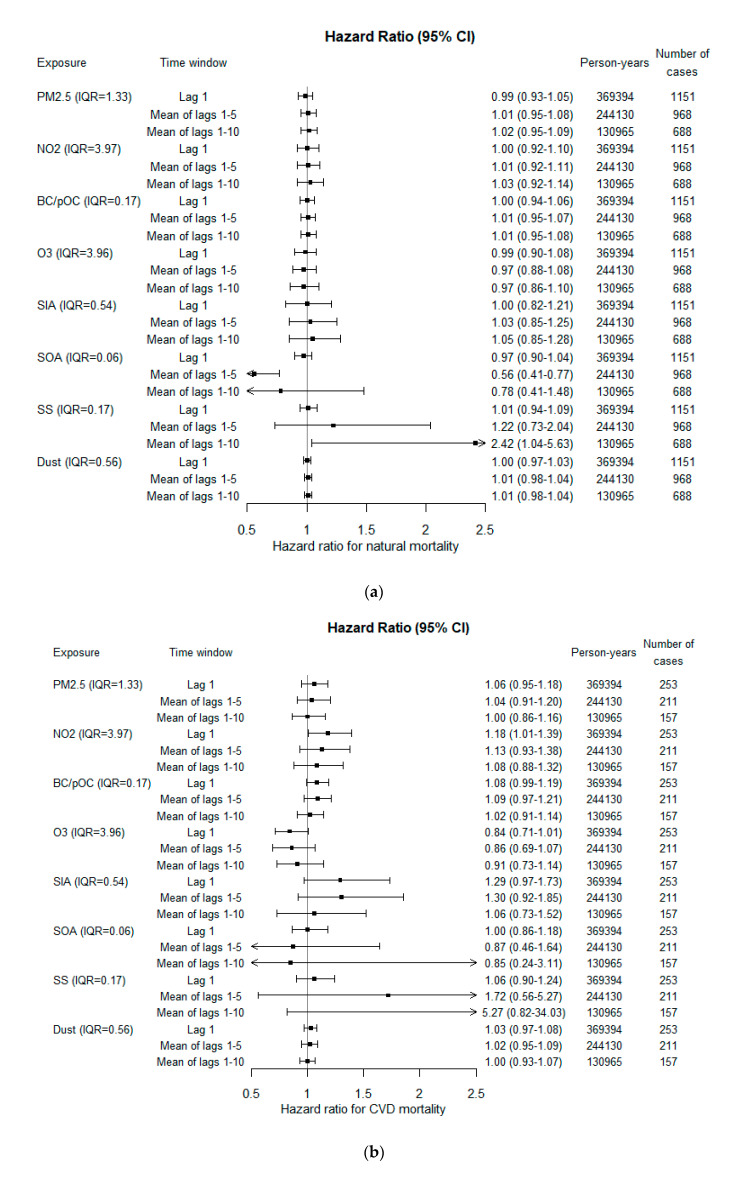

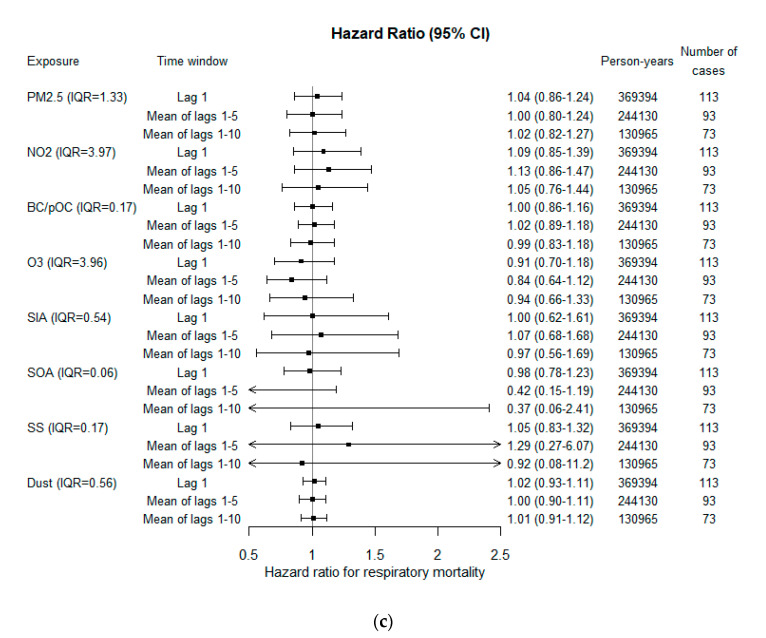
Adjusted hazard ratios for (**a**) natural, (**b**) CVD and (**c**) respiratory mortality per inter-quartile range (IQR). SS = sea salt, SOA = Secondary Organic Aerosols, SIA = Secondary Inorganic Aerosols, O_3_ = ozone, BC/pOC = primary emitted Carbonaceous Particles, NO_2_ = nitrogen dioxide, and PM_2.5_ = particulate matter with aerodynamic diameter ≤ 2.5 µm.

An indication of increase in natural mortality was observed in relation to concentrations of PM_2.5_, NO_2_, BC and pOC, SIA, SS and mineral dust (Figure 2a). The precision was, however, low, and none of these increased risks reached statistical significance. The risk increase associated with lag 1–10 exposures were 2% (95% CI −5–9%) per IQR for PM_2.5_, 3% (95% CI −8–14%) per IQR for NO_2_, 1% (95% CI −5–8%) for BC and pOC, 5% (95% CI −15–28%) for SIA, 142% (95% CI 4–463%) for SS and 1% (95% CI −2–4%) for dust. Higher hazard ratios were observed for lag 1–5 and highest for lag 1–10, compared with lag 1. This was, however, not the case for CVD mortality, where higher risks were observed in association with exposures closer in time (Figure 2b). For PM_2.5_ and NO_2_, the risk increase for CVD mortality was largest in relation to lag 1 exposure, where a statistically significant risk increase was found in relation to NO_2_. The risk increases associated with PM_2.5_ was 6% (95% CI −5–18%) per IQR and for NO_2_ 18% (95% CI 1–39%). For BC and pOC, the highest increased risk was observed in relation to lag 1–5 exposure; 9% (95% CI −3–21%) per IQR. Lag 1 exposures to SIA, SS and dust were observed to increase CVD risk by 29% (95% CI −3–73%), 6% (95% CI −10–24%) and 3% (95% CI −3–8%), respectively. High risk estimates were observed for lag 1–5 and 1–10 exposures to SS, 72 and 427% increased risks per IQR. For respiratory mortality, similar hazard ratio estimates as for natural mortality were observed except for a higher risk increase in relation to lag 1–5 NO_2_ exposure and no increased risk in relation to lag 1–10 exposure to SS (Figure 2c). A 13% (95% CI −14–47%) increased risk was observed per IQR NO_2_ and an 8% (95% CI −92–1020%) risk reduction per IQR SS.

For all mortality outcomes, adjustment for confounders increased the risk associated with BC and pOC and also stabilized the risk estimates for O_3_ between time windows of exposures. This was observed both for the same person-years and when also including individuals with missing information on confounders (Figure S2a–c and Figure S3a–c).

In two-pollutant models of mutual adjustment between PM_2.5_, NO_2_, BC and pOC and O_3_, the risk estimates for natural mortality associated with PM_2.5_ were reduced by adjusting for NO_2_ (Table S3). For BC and pOC the risk estimate increased after adjustment for NO_2_ at lag 1–5, but was reduced for lag 1–10 with the association with NO_2_ remaining. When adjusting risk estimates for SIA, SOA and SS by PM_2.5_, the associations with SOA and SS remained, however not the risk increase associated with SIA (Figure S4a–c). The associations with SOA did not remain in two-pollutant models including SIA (Figure S4a–c).

## 4. Discussion

In this study, increased mortality from CVD was associated with higher concentrations of NO_2_. Risk estimates of the same magnitude were also observed for natural and respiratory mortality, however with less precision. Increased risks were also indicated in relation to PM_2.5_, and higher for CVD mortality. For O_3_, inverse associations were observed for CVD and respiratory mortality. For the PM components, increased risk estimates were observed with higher BC and pOC, SIA and SS, but only the risk increase in relation to SS was found statistically significant. Decreased risks of mortality were, however, found with higher concentrations of SOA. The effect on natural mortality from an IQR increase was higher for SIA and SS, and on CVD and respiratory mortality also for NO_2_ and, in addition, on CVD mortality for BC and pOC.

The results on PM_2.5_, NO_2_, BC and pOC and O_3_ are in line with recently published results from the Danish Diet, cancer and health cohort [21]. However, the current study found a 7% increased risk per 5 µg/m^3^ lag 1–10 PM_2.5_ for natural mortality, which is lower than the Danish study estimate of 13% (95% CI 5–21%) risk increase in relation to lag 1–15 exposure. Both studies reported 7% increased risk per 10 µg/m^3^ NO_2_, 9% per 1 µg/m^3^ BC and pOC, and 7–8% decreased risk per 10 µg/m^3^ O_3_. For CVD mortality, the risk estimate for NO_2_ was higher in the current study, but lower for PM_2.5_. In addition, for respiratory mortality the risk estimate for NO_2_ was higher, but similar for PM_2.5_. A nested case-control registry study on the entire Danish population found lower risk increases for natural mortality; 4% (95% CI 2.0–6.3%) per 5 µg/m^3^ lag 1–5 PM_2.5_, 5% (95% CI 4–6%) per 10 µg/m^3^ lag 1–5 NO_2_, 5% (95% CI 2–8%) per µg/m^3^ lag 1–5 BC and a lower decreased risk of 4% (95% CI 3–5%) per 10 µg/m^3^ lag 1–5 O_3_ [39].

The findings for mortality associated with PM_2.5_ have been heterogeneous, however the meta-estimate among 22 cohorts within the European ESCAPE study showed a 7% (95% CI 2–13%) increased risk of natural mortality and 21% (95% CI −13–69%) increased risk of death in cerebrovascular disease per 5 µg/m^3^. The assessment of outdoor residential exposures was in these studies less detailed and was only assessed for the year of recruitment, which was varying between 1985 and 2007 for the different cohorts in the meta-analysis, and made long back extrapolation from measurements 2008–2011 needed for some cohorts. No association was found in relation to death in ischemic heart disease or myocardial infarction. Within earlier studies, a review and meta-analysis by Hoek et al. (2013) [3] found 6% (95% CI 4–8%) and 15% (95% CI 4–27%) increased risks of all-cause mortality and cardiovascular mortality per 5 µg/m^3^, respectively. These previous results of a stronger association between PM and mortality with cardiovascular causes compared to all natural causes of mortality have also been reported by others [44], and are in agreement with our findings on CVD mortality and most apparent in relation to exposures during the last 5 years. As concluded, the cause-specific analyses from ESCAPE only reported increased risk estimates for mortality in cerebrovascular disease, but not for death in ischemic heart disease or myocardial infarction, and no association was found in relation to the composite CVD mortality outcome [45]. In the review by Hoek et al. (2013) [3], elemental carbon was found associated with a 6% (95% CI 5–7%) increased risk of all-cause mortality per µg/m^3^.

Only an extremely limited number of studies on long-term concentrations of PM components and mortality have been published. Taken together, these studies show that it is difficult to disentangle the independent effect of any of the investigated components from the effect of PM_2.5_ mass. For this reason, WHO, US EPA and others have not proposed any concentration-response functions for specific elements or sources.

A systematic review of health effects related to long-term exposure to fine particulate matter components and health recently reported meta-estimate risk increases per IQR (median among included studies) for non-accidental premature mortality by 1.78% (95% CI 0.36–3.22%) for BC, 2.6% (95% CI 0.88–4.34%) for nitrate, 9.4% (95% CI 6.33–12.56%) for Zn and 5.9% (95% CI 3.25–8.61%) for Si [19]. Fe, nitrate, Zn and Si were also found associated with cardiovascular mortality. This risk estimate for BC was lower than the risk estimate for BC and pOC in our study, 3.0 compared with 6.0% increased risk per µg/m^3^. The Danish diet, cancer and health study reported increased risks for all-cause, CVD and respiratory mortality associated with both SOA and SIA [22]. The risk increase per IQR for SOA was 8% (95% CI 3–13%) for all-cause mortality, whereas decreased risks were observed in our study. For SIA, the risk increase per µg/m^3^ was in general higher in our study, with all-cause mortality at 9.5% (95% CI −26–58%) for lag 1–10 exposure compared with 3.9% (95% CI −3.8–12%) for lag 1–15 in their study. SS did not increase the risk for mortality in the Danish study. The effects of long-term exposure to sea salt (or Na and Cl) on mortality are not further known from cohort studies. However, studies of short-term concentrations and the daily number of deaths have shown a positive association both in single locations, multi-city studies and meta-analysis [46,47,48].

Within the large US Medicare population, a PM_2.5_ composition with a higher concentration of sulfate, nitrate and pOC was associated with a lower HR for all-cause mortality, whereas an increase in Al, Ca, Cu, EC, Fe or V was associated with an increased HR [49]. Limited changes in HRs were observed after accounting for Ni and Zn. In another US cohort, ischemic heart disease mortality was found associated with about a five times higher risk increase for the coal combustion-related part of PM_2.5_ than for total PM_2.5_ mass concentration [50]. Black carbon (or soot) related to diesel traffic was also associated with IHD mortality, but not wind-blown soil or biomass combustion. In another US Medicare study, one-standard deviation increases in 7-year average EC, Si and NO_3_^−^ concentrations were associated with 1.3% (95% posterior interval (PI) 0.3–2.2%), 1.4% (95% PI 0.6–2.4%) and 1.2% (95% PI 0.4–2.1%) increases in monthly mortality, respectively, adjusting for total PM_2.5_ the year before [51]. The ESCAPE study also reported an association between PM_2.5_ sulfur and all-cause mortality independent of PM_2.5_ mass exposure [52]. In a pooled analysis of eight of the European ESCAPE cohorts, residential exposure to the 2010 annual average concentration of eight PM_2.5_ components (Cu, Fe, K, Ni, S, Si, V and Zn) was estimated with Europe-wide models at a 100 × 100 m scale, and all were found associated with all-cause mortality [20]. In two pollutant models, adjusting for PM_2.5_ or NO_2_, Ni, S, Si, V and Zn remained associated with all-cause mortality. As part of the National Particle Component Toxicity (NPACT) Initiative, the review panel concluded that PM_2.5_ and elemental carbon was associated with all-cause mortality, and EC also with ischemic heart disease mortality [53]. No support for an association with organic carbon was found. Additionally, in the US, within the California Teacher’s study, cardiopulmonary disease mortality was found in association with NO_3_^−^ and SO_4_^2−^. Increased risk of ischemic heart disease mortality was found in association with EC, pOC, SO_4_^2−^, NO_3_^−^ and SOA [11,15].

The modelled concentrations of SOA are in the DEHM model based on a volatility basis set approach [54] and a state-of-art parameterisation describing the emissions of biogenic volatile organic compounds [30]. The latter is highly dependent on vegetation types and on a number of meteorological and local parameters, and as such carries some uncertainty. The contribution of SOA to PM_2.5_ is relatively small, and the spatial gradient is also small. Overall, both the temporal and spatial variations of the SOA concentrations are encumbered with considerable uncertainty. Sea salt is also based on a parameterisation commonly used in air pollution modelling, where the formation of sea salt aerosols at the marine surface is a function of wind speed, sea surface temperature and salinity of the water (see [29] for details). Both components have been evaluated against relevant observations and the model captures the overall distribution and variation.

Possible explanations to the observed heterogeneity of PM associated risk for premature mortality between studies include differences in exposure assessment with differences in geographical resolution, particle composition, population characteristics, exposure windows, housing with differences in particle infiltration and the ability to adjust for confounders. Meta-analysis results of epidemiological studies on health outcomes have shown that a higher precision in the exposure assessment results in higher relative risk estimates [1]. Higher relative risks have also been found in relation to local compared with distal sources, also indicating that high spatial resolution exposure data is of importance to successfully model differences in mortality [24]. The studies by Hvidtfeldt et al. (2019a and b) [21,22] used the same exposure models DEHM/UBM as in the present study, but with a higher resolution in the regional contribution and combined with address-level AirGIS information, resulting in address-level modelled concentrations. Both studies generally provided similar hazard ratio estimates for natural mortality in relation to NO_2_ and BC and pOC, but the hazard ratio in relation to PM_2.5_ was lower in the current study. Both the current and the Danish studies used moving averages based on annual mean concentrations at the place of residence, which may be a more important measure of exposure in relation to mortality compared with residential concentrations at the year of recruitment or time-weighted average exposures during the follow-up. These studies also adjusted for individual questionnaire and registry data on potential confounders and neighbourhood level data on socioeconomics. In studies of air pollution effects, failure to adjust for such covariates often also results in bias towards the null.

### Strengths and Limitations

Air pollution assessments for exposure studies can be derived in a number of more or less complex ways. Today the required air pollution data are typically based on various combinations of observations (e.g., satellites) and/or modelling such as the land-use regression hybrid models used for the US [55,56] and for Europe in the ELAPSE project [57]. Here, we apply a state-of-the-art chemistry-transport model system, which can be an advantage when covering many years such as in the current study. The limitation is that the peak exposure at a busy street, for example, cannot be captured with the 1 km × 1 km resolution applied. The 1 km × 1 km data can, however, be a reasonable proxy for the average exposure a person experiences when living and moving around in an urban area. Several recent studies have applied a similar setup with respect to exposure data resolution (e.g., [39,55]). Exposure misclassifications at the individual level will arise since the true personal exposure not only include exposures at the place of residence, but for instance also at the workplace and during the commute to and from work. Assuming that the difference between modelled residential exposure and other exposures are non-differential in terms of mortality, this exposure misclassification would create a bias towards the null. If individuals susceptible to developing diseases would tend to move away from highly polluted areas, and there is a lag of some years before the effect occurred among less susceptible, this would also cause such a bias. In addition, non-participation when recruiting individuals to the cohort may underestimate the true air pollution effect on mortality in the target population if non-participation related to low socioeconomic status, which in turn is related to increased risk for pre-term mortality, also relate to higher air pollution exposure levels. However, if a low socioeconomic status is related to lower exposure levels this would overestimate the true association. During the years of follow up there has been a decreasing trend in both total concentrations of PM and age specific mortality. Even though adjustments were made for calendar year in the Cox models there is a risk that we were not able to distinguish between time trend and air pollution effects. Limitations also include the lack of information on green space near the home address which could be a confounder or effect-modifier of air pollution effects, and we also lacked noise data.

As previously stated, another strength of this study was the ability to adjust for a large set of confounders at both the individual and area level, however information on confounders from questionnaires were only available at baseline. Even though we expect the cohort participants with ages ranging from 40 years to have fairly stable lifestyles and habits, several things may affect such baseline variables, such as incidence of disease during follow up. Additionally, the intervention by health screening may affect such habits.

This study additionally contributes with hazard ratio estimates between PM and mortality in the lower range of exposure, with PM_2.5_ (with a few exceptions) ranging between 3 to 10 μg/m^3^. Even though this is below the World Health Organization (WHO) guideline of 10 μg/m^3^, and considerably lower than current European Union standard of 25 μg/m^3^, the study generally found increased risks for premature mortality.

## 5. Conclusions

This Swedish cohort study with relatively low exposures showed increased risks of mortality in association with long-term exposure NO_2_, and indications of increased risks in relation to PM_2.5_, BC and pOC and SIA, and thus support long-term air pollution-associated effects. Since risk increases were observed even at relatively low exposures, the findings are relevant for future decisions concerning air quality policies.

## Figures and Tables

**Table 1:** Descriptive statistics of the study participants.

		All	In Tertiles of PM_2.5_		
			<4.36 µg/m^3^	4.36–4.81 µg/m^3^	>4.81 µg/m^3^
Participants (n)		43,216	14,261	14,261	14,694
Women		52%	50%	52%	53%
BMI (kg/m^2^; mean ± sd)		25.5 (4.1)	26.0 (4.4)	25.4 (4.0)	25.1 (3.9)
Smoking status	Current smoker	20%	15%	21%	25%
	Former smoker	30%	28%	31%	30%
	Never smoker	49%	56%	47%	44%
	Missing data	1%	1%	1%	2%
Leisure time physical activity	Sedentary	36%	33%	38%	37%
	Moderate	42%	37%	42%	45%
	Intermediate and vigorous	21%	28%	19%	16%
	Missing data	2%	2%	1%	2%
Alcohol consumption	Daily	1%	1%	1%	1%
	Weekly	16%	17%	18%	14%
	Seldom	42%	48%	46%	33%
	Never	2%	3%	1%	0%
	Missing data	39%	31%	34%	52%
Low fruit intake	Yes	5%	7%	4%	3%
	Missing	15%	3%	9%	34%
Low vegetable intake	Yes	4%	5%	4%	4%
	Missing	16%	4%	10%	34%
Married/living with partner	Yes	76%	78%	77%	75%
	Missing data	1%	1%	1%	1%
Education level	Primary school or less	31%	21%	33%	38%
	Up to secondary school or equivalent	30%	35%	29%	25%
	University degree and more	39%	43%	38%	36%
	Missing data	1%	1%	1%	1%
Occupation	Gainfully employed	85%	87%	85%	83%
	Unemployed/not gainfully employed	6%	6%	6%	7%
	Retired	4%	5%	5%	3%
	Missing data	4%	2%	4%	7%

**Table 2:** Air pollution concentrations for the years of follow-up presented as means and standard deviations together with quartile limits and inter-quartile ranges (IQRs).

	Mean (sd)	Median (1st Quartile–3rd Quartile)	IQR
PM_2.5_	4.90 (1.92)	4.55 (3.81–5.14)	1.33
NO_2_	7.09 (3.49)	6.63 (4.82–8.79)	3.97
BC/pOC	0.66 (0.25)	0.62 (0.54–0.71)	0.17
O_3_	50.76 (3.27)	50.93 (48.96–52.92)	3.96
SIA	1.67 (0.43)	1.73 (1.33–1.87)	0.54
SOA	0.23 (0.05)	0.23 (0.20–0.26)	0.06
SS	1.02 (0.12)	1.00 (0.95–1.12)	0.17
dust	1.32 (1.54)	0.98 (0.73–1.29)	0.56

SS = sea salt, SOA = Secondary Organic Aerosols, SIA = Secondary Inorganic Aerosols, O_3_ = ozone, BC/pOC = primary emitted Carbonaceous Particles, NO_2_ = nitrogen dioxide, and PM_2.5_ = particulate matter with aerodynamic diameter ≤ 2.5 µm.

## Data Availability

No additional data available.

## Supplementary Materials

The following are available online at https://www.mdpi.com/article/10.3390/ijerph18168476/s1, Table S1: The coefficient of variation (CV, the standard deviation in relation to the mean) differed between calendar years. Table S2a. Pearson correlation coefficients between lag 1 exposures. Table S2b. Pearson correlation coefficients between lag 1–5 exposures. Table S2c. Pearson correlation coefficients between lag 1–10 exposures. Table S3. Adjusted hazard ratios for natural mortality in two-pollutant models, and their corresponding single pollutant result. Figure S1a. Example of the modelled distribution of the annual mean PM_2.5_ for 2014. Figure S1b. An example of the modelled distribution of the annual mean NO_2_ for 2014. Figure S1c. An example of the modelled distribution of the annual mean BC/pOC for 2014. Figure S1d. An example of the modelled distribution of the annual mean O_3_ for 2014. Figure S1e. An example of the modelled distribution of the annual mean SIA for 2014. Figure S1f. An example of the modelled distribution of the annual mean SOA for 2014. Figure S1g. An example of the modelled distribution of the annual mean sea salt for 2014. Figure S1h. An example of the modelled distribution of the annual mean anthropogenic mineral dust for 2014. Figure S2a. Unadjusted hazard ratios for natural mortality per inter-quartile range (IQR), also including individuals with missing information on covariates. Figure S2b. Unadjusted hazard ratios for CVD mortality per inter-quartile range (IQR), also including individuals with missing information on covariates. Figure S2c. Unadjusted hazard ratios for respiratory mortality per inter-quartile range (IQR), also including individuals with missing information on covariates. Figure S3a. Unadjusted hazard ratios for natural mortality per inter-quartile range (IQR), excluding individuals with missing information on covariates. Figure S3b. Unadjusted hazard ratios for CVD mortality per inter-quartile range (IQR), excluding individuals with missing information on covariates. Figure S3c. Unadjusted hazard ratios for respiratory mortality per inter-quartile range (IQR), excluding individuals with missing information on covariates. Figure S4a. Adjusted hazard ratios for natural mortality per inter-quartile range (IQR) in two-pollutant models; SIA, SOA and SS adjusted for total PM_2.5_, and mutually adjusted estimates for SIA and SOA. Figure S4b. Adjusted hazard ratios for CVD mortality per inter-quartile range (IQR) in two-pollutant models; SIA, SOA and SS adjusted for total PM_2.5_, and mutually adjusted estimates for SIA and SOA. Figure S4c. Adjusted hazard ratios for respiratory mortality per inter-quartile range (IQR) in two-pollutant models; SIA, SOA and SS adjusted for total PM_2.5_, and mutually adjusted estimates for SIA and SOA.
