# Supplementary material for: Long-Term Residential Exposure to Particulate Matter and Its Components, Nitrogen Dioxide and Ozone—A Northern Sweden Cohort Study on Mortality
Source: Int J Environ Res Public Health. 2021 Aug 11;18(16):8476. doi: 10.3390/ijerph18168476 (PMC8393394; doi:10.3390/ijerph18168476)
Supplement: Supplementary file 1 [file ijerph-18-08476-s001.zip › ijerph-1063294-supplementary.pdf]

International Journal of
Environmental Research and Public Health

MDPI

Supplementary Materials

Table S1. The coefficient of variation (CV, the standard deviation in relation to the mean) differed between calendar years (as described in the table below).

	Min.	1st Qu.	Median	Mean	3rd Qu.	Max.
PM2.5	0.22	0.29	0.37	0.34	0.40	0.45
NO2	0.46	0.48	0.52	0.51	0.54	0.60
BC/pOC	0.26	0.32	0.39	0.40	0.46	0.61
O3	0.05	0.06	0.07	0.07	0.08	0.09
SIA	0.01	0.15	0.22	0.19	0.24	0.30
SOA	0.02	0.06	0.08	0.08	0.09	0.17
Sea salt	0.01	0.05	0.09	0.08	0.10	0.13
Dust	0.65	0.89	1.12	1.02	1.20	1.26

Table S2a. Pearson correlation coefficients between lag 1 exposures.

	PM2.5	NO2	BC/pOC	O3	SIA	SOA	SS	dust
PM2.5	1.00	0.78	0.85	-0.67	0.47	0.23	0.10	0.96
NO2	0.78	1.00	0.82	-0.94	0.33	0.17	0.06	0.74
BC/pOC	0.85	0.82	1.00	-0.77	0.38	0.18	-0.05	0.79
O3	-0.67	-0.94	-0.77	1.00	-0.29	0.01	0.11	-0.64
SIA	0.47	0.33	0.38	-0.29	1.00	0.31	0.07	0.23
SOA	0.23	0.17	0.18	0.01	0.31	1.00	0.10	0.13
SS	0.10	0.06	-0.05	0.11	0.07	0.10	1.00	0.03
Dust	0.96	0.74	0.79	-0.64	0.23	0.13	0.03	1.00

Table S2b. Pearson correlation coefficients between lag 1–5 exposures.

	PM2.5	NO2	BC/pOC	O3	SIA	SOA	SS	dust
PM2.5	1.00	0.80	0.87	-0.72	0.40	0.39	0.24	0.97
NO2	0.80	1.00	0.84	-0.98	0.29	0.30	0.17	0.77
BC/pOC	0.87	0.84	1.00	-0.80	0.34	0.35	0.16	0.82
O3	-0.72	-0.98	-0.80	1.00	-0.25	-0.19	-0.15	-0.69
SIA	0.40	0.29	0.34	-0.25	1.00	0.71	0.55	0.19
SOA	0.39	0.30	0.35	-0.19	0.71	1.00	0.09	0.25
SS	0.24	0.17	0.16	-0.15	0.55	0.09	1.00	0.13
Dust	0.97	0.77	0.82	-0.69	0.19	0.25	0.13	1.00

Table S2c. Pearson correlation coefficients between lag 1–10 exposures.

	PM2.5	NO2	BC/pOC	O3	SIA	SOA	SS	dust
PM2.5	1.00	0.82	0.88	-0.76	0.27	0.36	0.21	0.98
NO2	0.82	1.00	0.85	-0.99	0.20	0.29	0.15	0.79
BC/pOC	0.88	0.85	1.00	-0.82	0.25	0.33	0.16	0.83
O3	-0.76	-0.99	-0.82	1.00	-0.21	-0.26	-0.14	-0.73
SIA	0.27	0.20	0.25	-0.21	1.00	0.75	0.55	0.10
SOA	0.36	0.29	0.33	-0.26	0.75	1.00	0.19	0.24
SS	0.21	0.15	0.16	-0.14	0.55	0.19	1.00	0.11
Dust	0.98	0.79	0.83	-0.73	0.10	0.24	0.11	1.00

Table S3. Adjusted hazard ratios for natural mortality in two-pollutant models, and their corresponding single pollutant result.

Model	Purpose	Exposure	Time window	Increment (μg/m3)	Multiple pollutant estimates	Single pollutant estimates
					HR (95% CI)	
1	Adjusted for O3	PM2.5	One year lag	5	0.92 (0.70-1.22)	0.97 (0.77-1.22)
		O3	One year lag	10	0.92 (0.70-1.22)	0.97 (0.77-1.21)
2	Adjusted for O3	NO2	One year lag	10	0.89 (0.54-1.49)	1.01 (0.81-1.26)
		O3	One year lag	10	0.87 (0.51-1.48)	0.97 (0.77-1.21)
3	Adjusted for O3	BC/pOC	One year lag	1	0.91 (0.51-1.62)	1.00 (0.70-1.43)
		O3	One year lag	10	0.92 (0.64-1.31)	0.97 (0.77-1.21)
4	Adjusted for NO2	PM2.5	One year lag	5	0.98 (0.92-1.05)	0.97 (0.77-1.22)

		NO2	One year lag	10	1.08 (0.78-1.49)	1.01 (0.81-1.26)
5	Adjusted for NO2	BC/pOC	One year lag	1	0.96 (0.51-1.80)	1.00 (0.70-1.43)
		NO2	One year lag	10	1.03 (0.71-1.52)	1.01 (0.81-1.26)
1	Adjusted for O3	PM2.5	Mean of lags 1–5	5	0.99 (0.69-1.40)	1.04 (0.82-1.32)
		O3	Mean of lags 1–5	10	0.92 (0.63-1.36)	0.93 (0.72-1.21)
2	Adjusted for O3	NO2	Mean of lags 1–5	10	0.30 (0.09-1.05)	1.02 (0.81-1.30)
		O3	Mean of lags 1–5	10	0.25 (0.06-1.01)	0.93 (0.72-1.21)
3	Adjusted for O3	BC/pOC	Mean of lags 1–5	1	0.92 (0.46-1.86)	1.06 (0.73-1.52)
		O3	Mean of lags 1–5	10	0.89 (0.55-1.44)	0.93 (0.72-1.21)
4	Adjusted for NO2	PM2.5	Mean of lags 1–5	5	1.01 (0.94-1.10)	1.04 (0.82-1.32)
		NO2	Mean of lags 1–5	10	0.97 (0.66-1.45)	1.02 (0.81-1.30)
5	Adjusted for NO2	BC/pOC	Mean of lags 1–5	1	1.09 (0.53-2.22)	1.06 (0.73-1.52)
		NO2	Mean of lags 1–5	10	0.97 (0.61-1.55)	1.02 (0.81-1.30)
1	Adjusted for O3	PM2.5	Mean of lags 1–10	5	1.06 (0.67-1.67)	1.07 (0.83-1.39)
		O3	Mean of lags 1–10	10	0.98 (0.57-1.68)	0.93 (0.68-1.26)
2	Adjusted for O3	NO2	Mean of lags 1–10	10	0.90 (0.12-6.61)	1.07 (0.81-1.40)
		O3	Mean of lags 1–10	10	0.83 (0.09-7.85)	0.93 (0.68-1.26)
3	Adjusted for O3	BC/pOC	Mean of lags 1–10	1	1.01 (0.47-2.15)	1.09 (0.75-1.58)
		O3	Mean of lags 1–10	10	0.93 (0.51-1.71)	0.93 (0.68-1.26)
4	Adjusted for NO2	PM2.5	Mean of lags 1–10	5	1.01 (0.91-1.13)	1.07 (0.83-1.39)
		NO2	Mean of lags 1–10	10	1.00 (0.57-1.73)	1.07 (0.81-1.40)
5	Adjusted for NO2	BC/pOC	Mean of lags 1–10	1	1.02 (0.46-2.27)	1.09 (0.75-1.58)
		NO2	Mean of lags 1–10	10	1.06 (0.60-1.86)	1.07 (0.81-1.40)

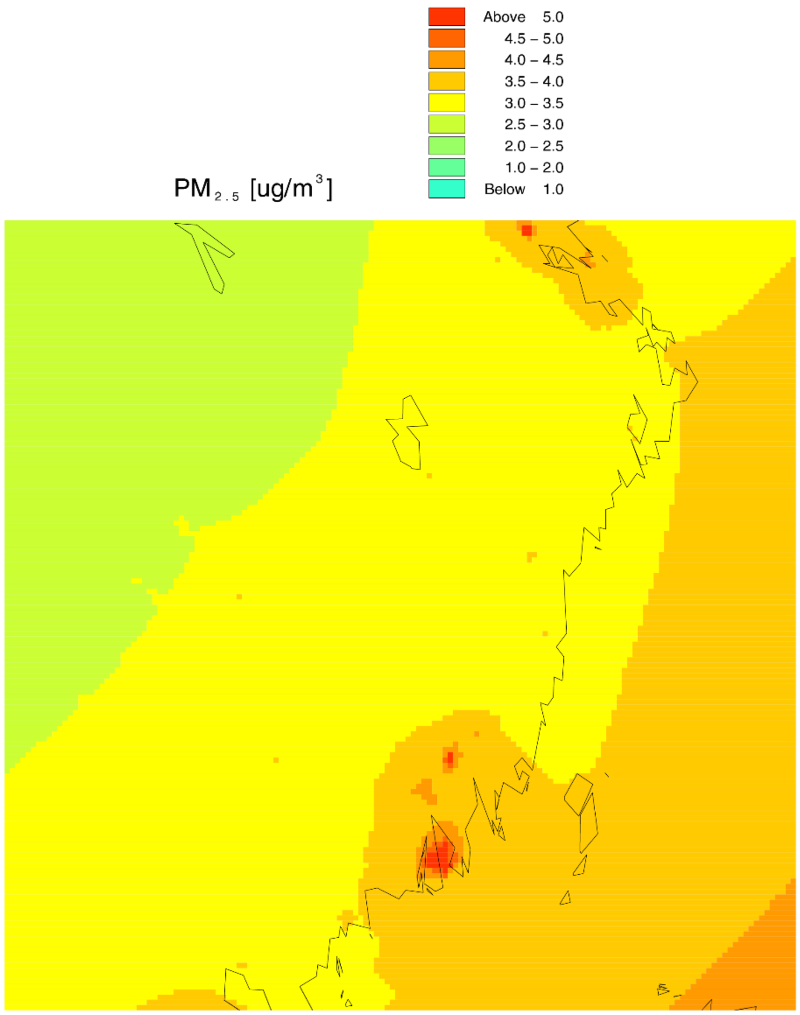

Figure S1a. Example of the modelled distribution of the annual mean $PM_{2.5}$ for 2014.

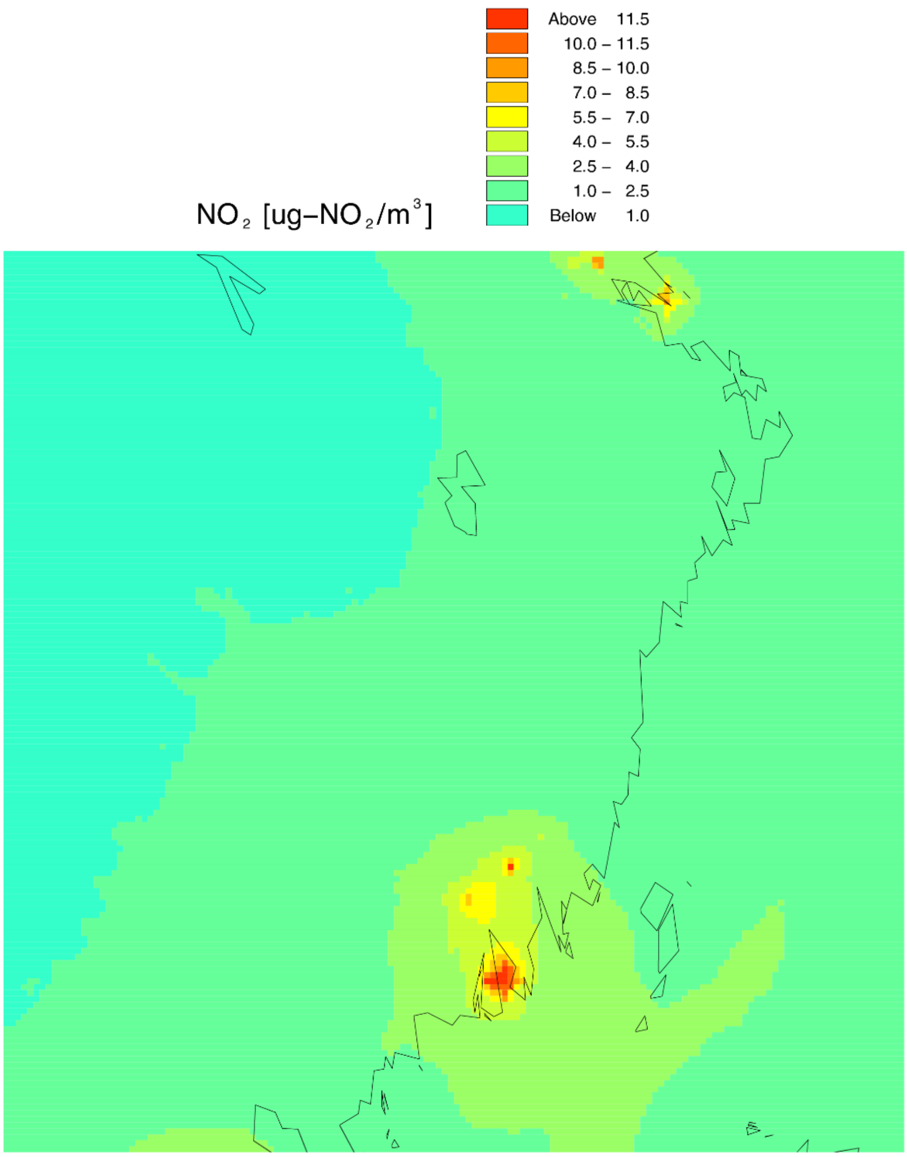

Figure S1b. An example of the modelled distribution of the annual mean NO_2 for 2014.

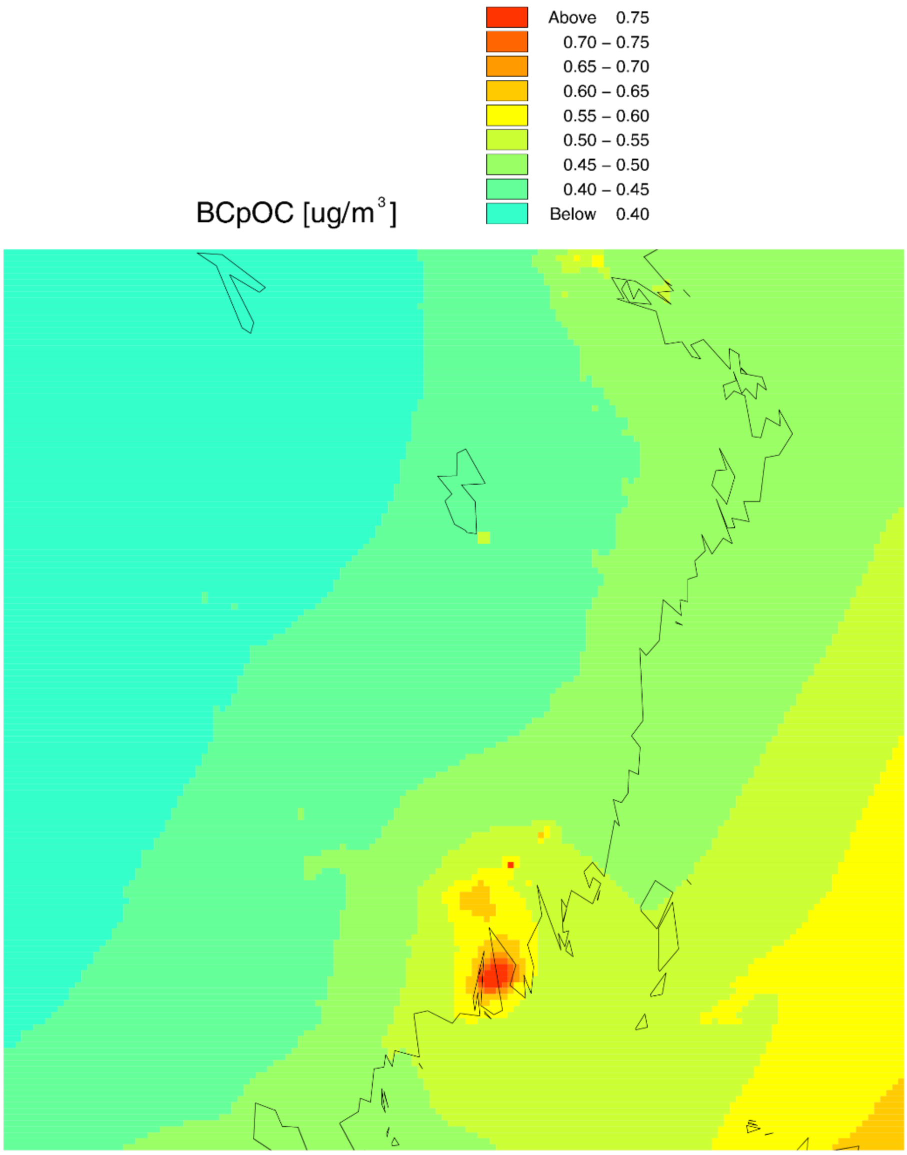

Figure S1c. An example of the modelled distribution of the annual mean BC/pOC for 2014.

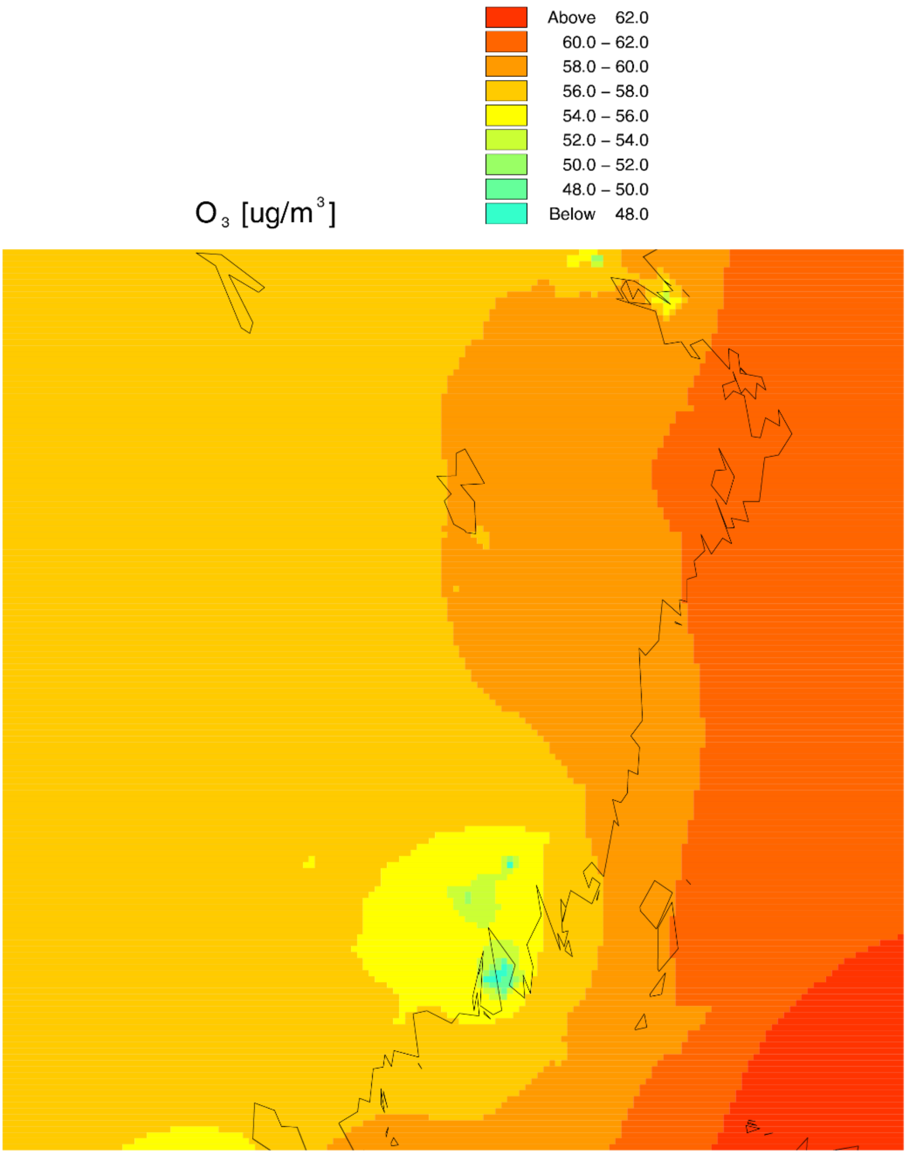

Figure S1d. An example of the modelled distribution of the annual mean O_3 for 2014.

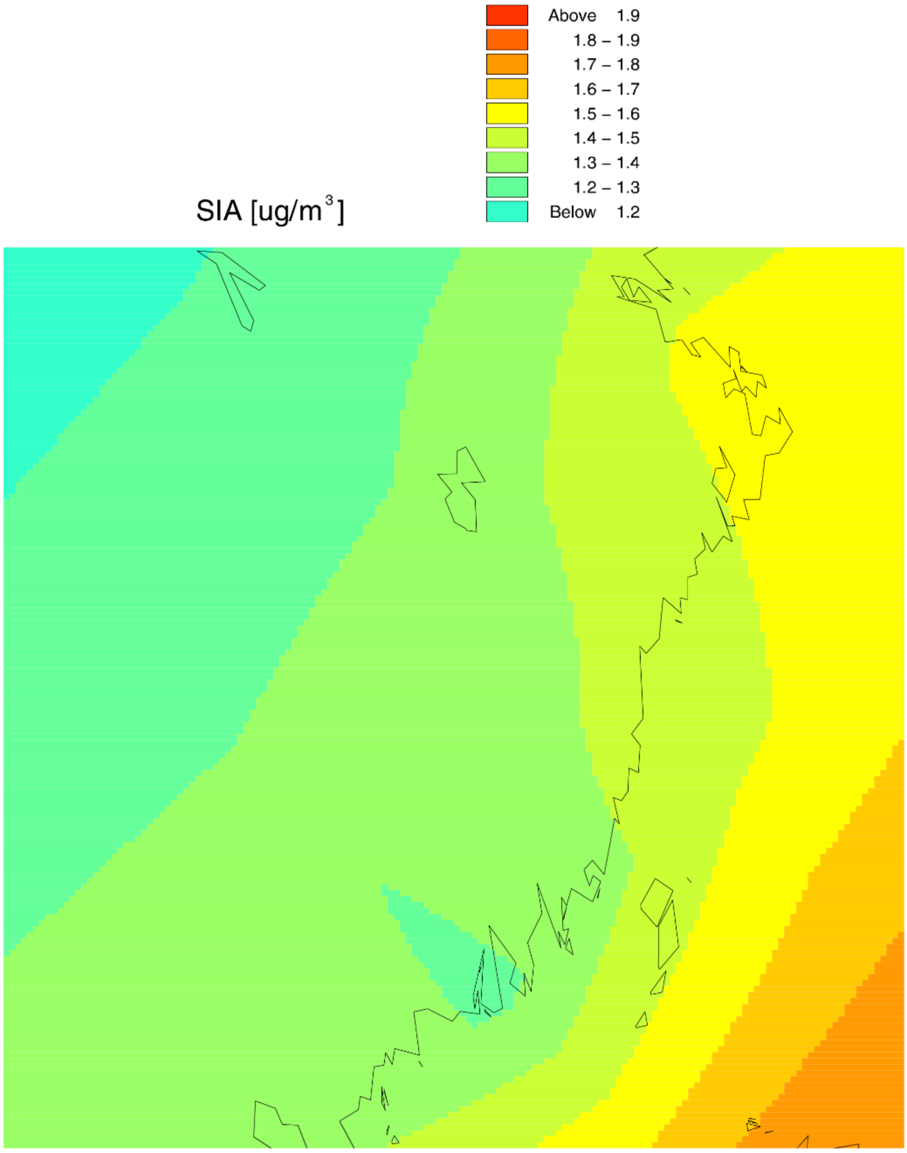

Figure S1e. An example of the modelled distribution of the annual mean SIA for 2014.

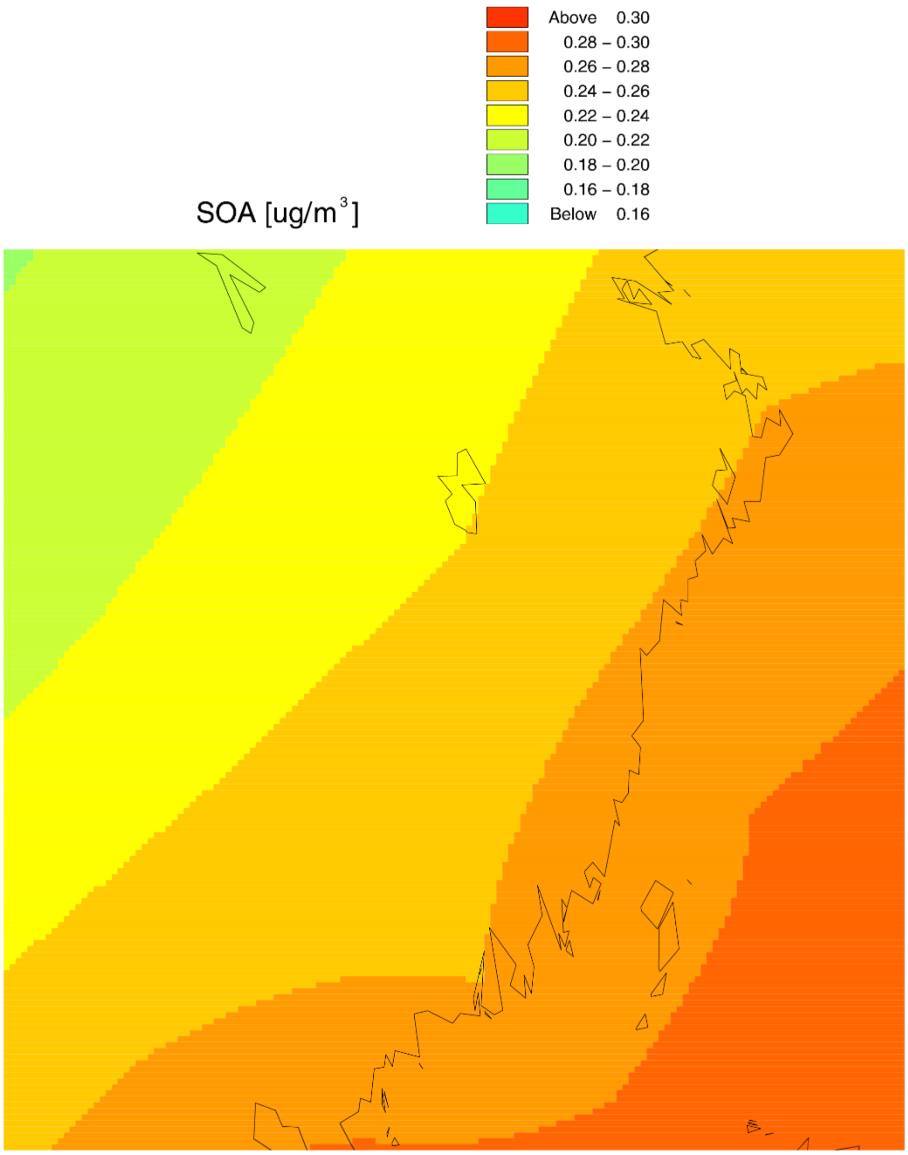

Figure S1f. An example of the modelled distribution of the annual mean SOA for 2014.

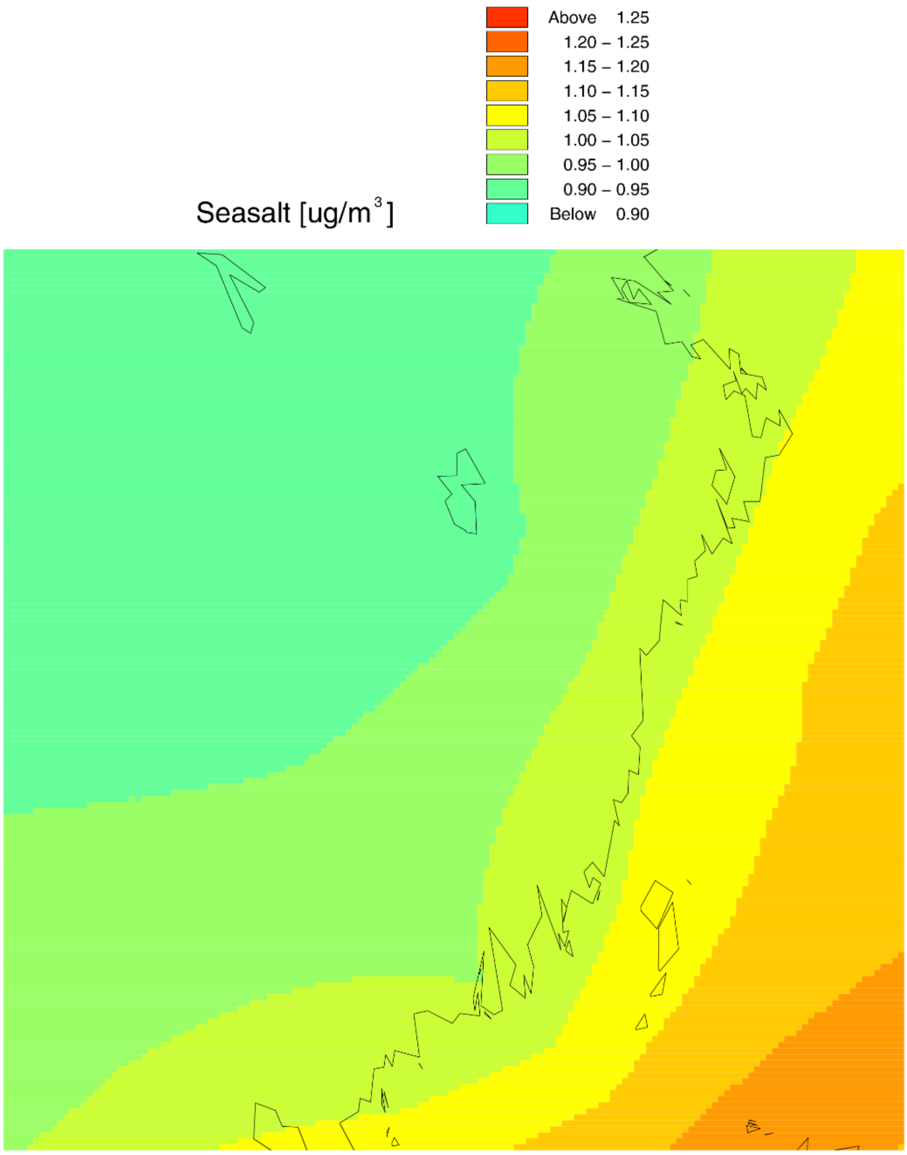

Figure S1g. An example of the modelled distribution of the annual mean sea salt for 2014.

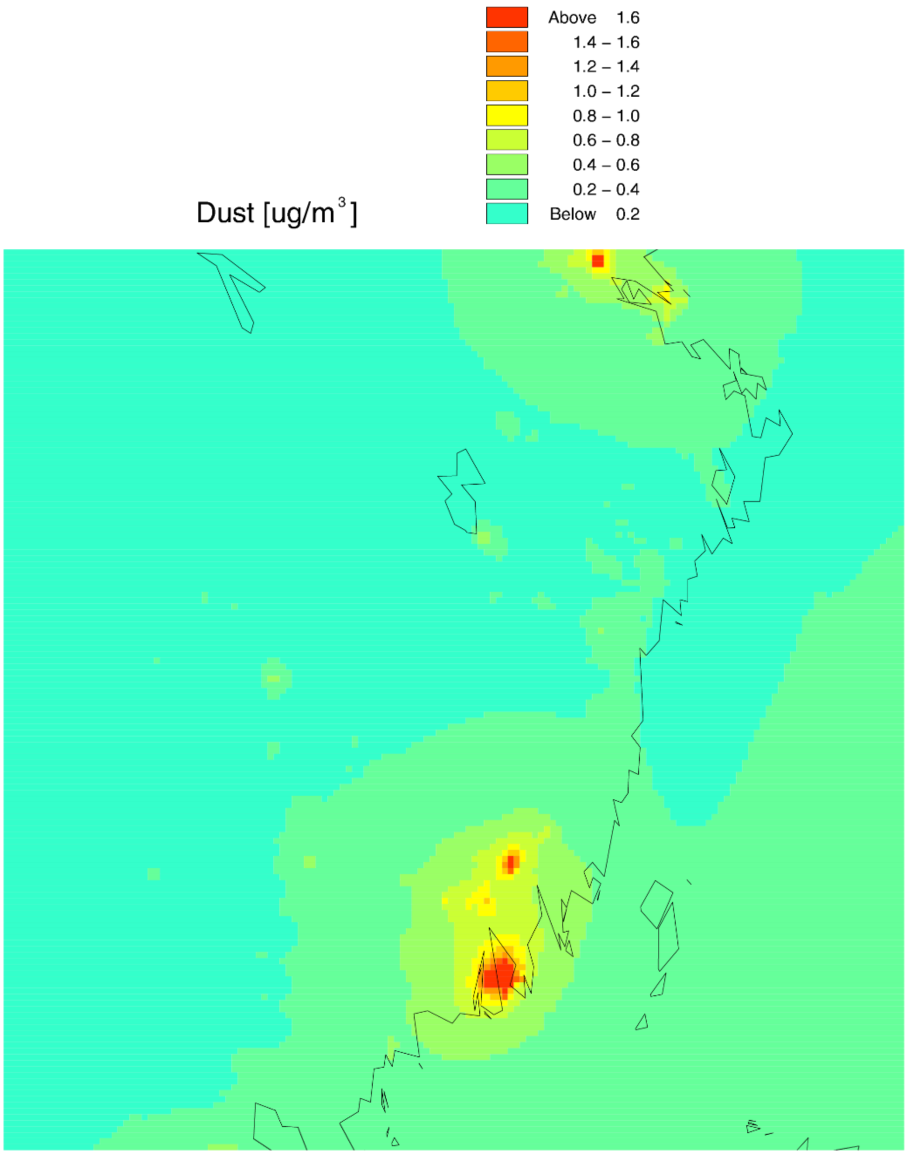

Figure S1h. An example of the modelled distribution of the annual mean anthropogenic mineral dust for 2014.

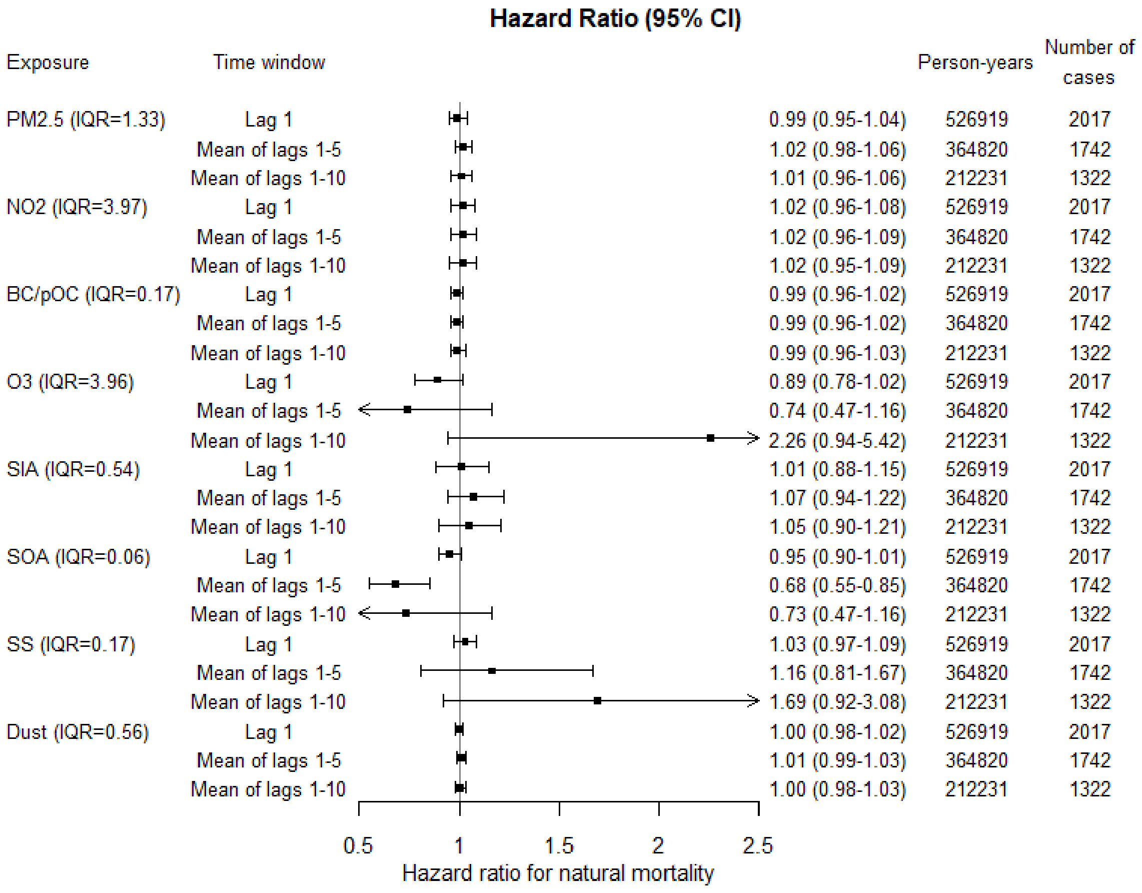

Figure S2a. Unadjusted hazard ratios for natural mortality per inter-quartile range (IQR), also including individuals with missing information on covariates.

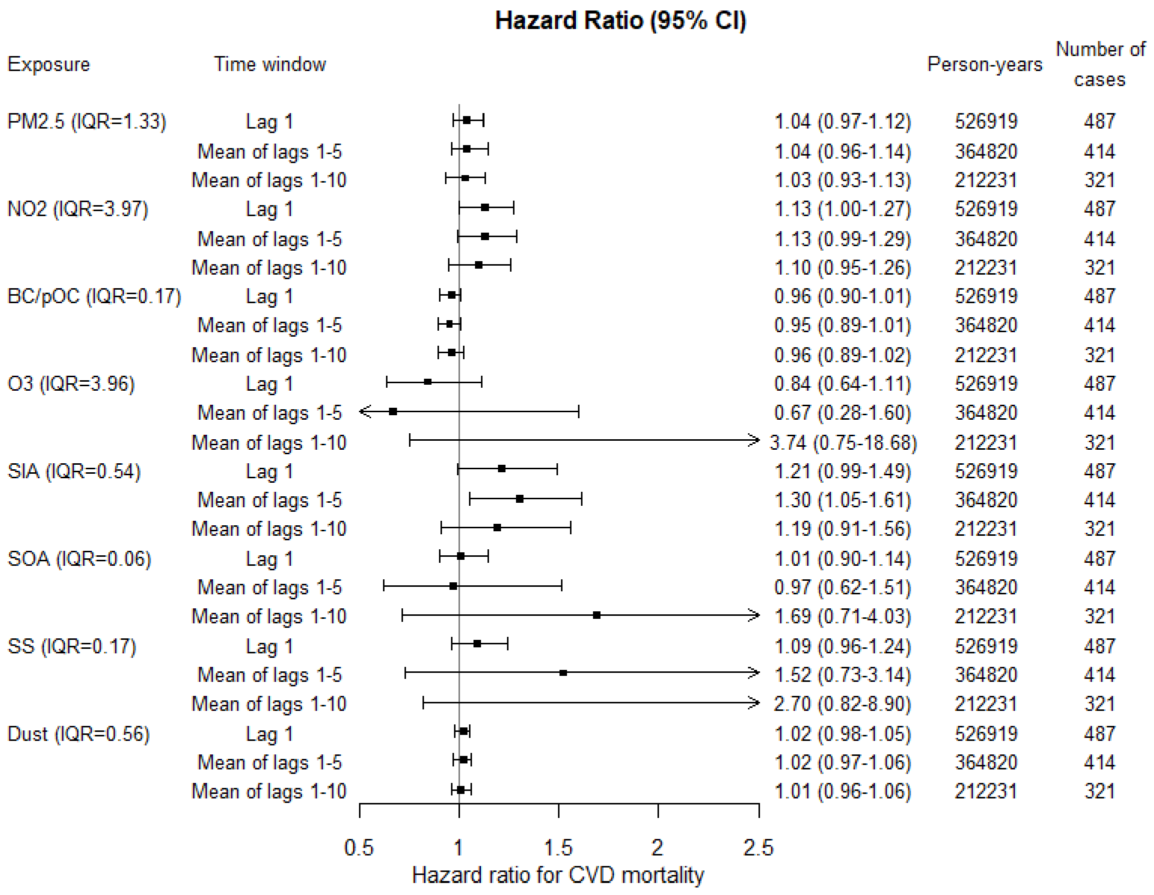

Figure S2b. Unadjusted hazard ratios for CVD mortality per inter-quartile range (IQR), also including individuals with missing information on covariates.

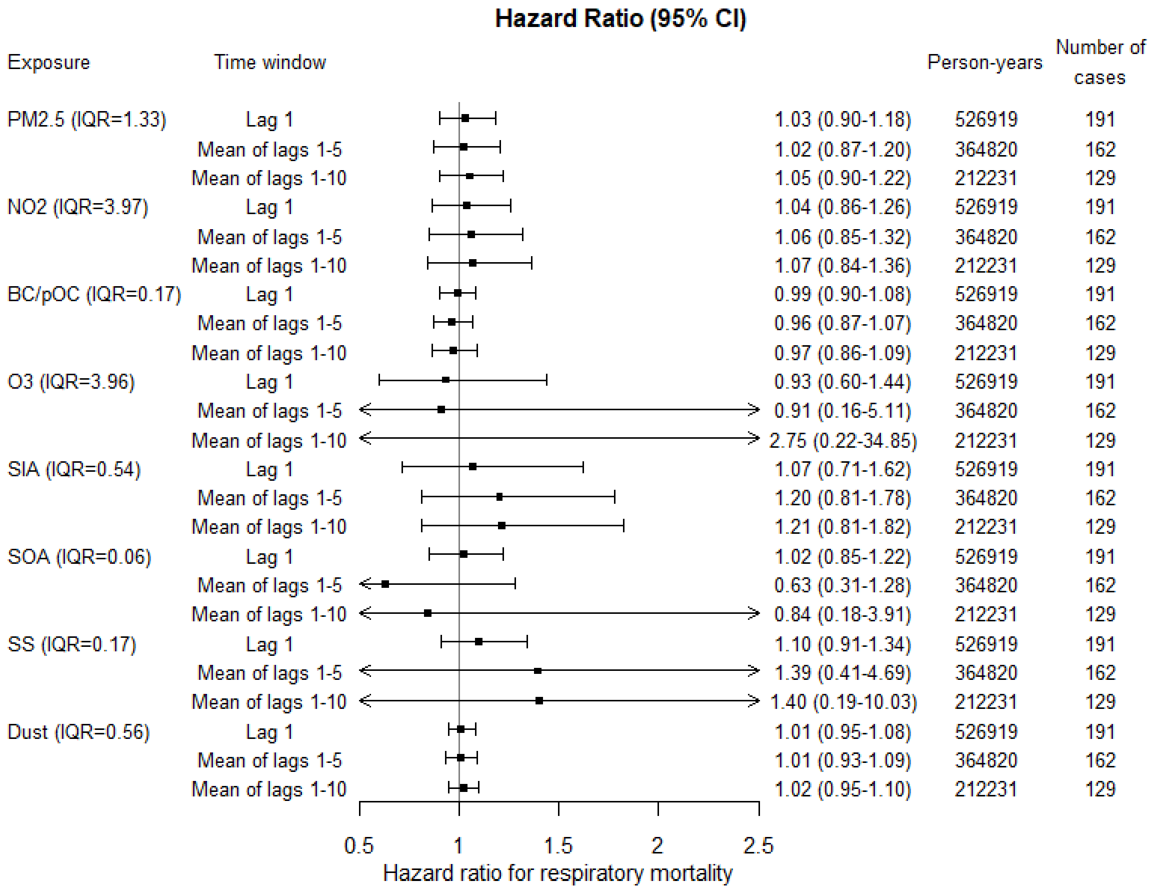

Figure S2c. Unadjusted hazard ratios for respiratory mortality per inter-quartile range (IQR), also including individuals with missing information on covariates.

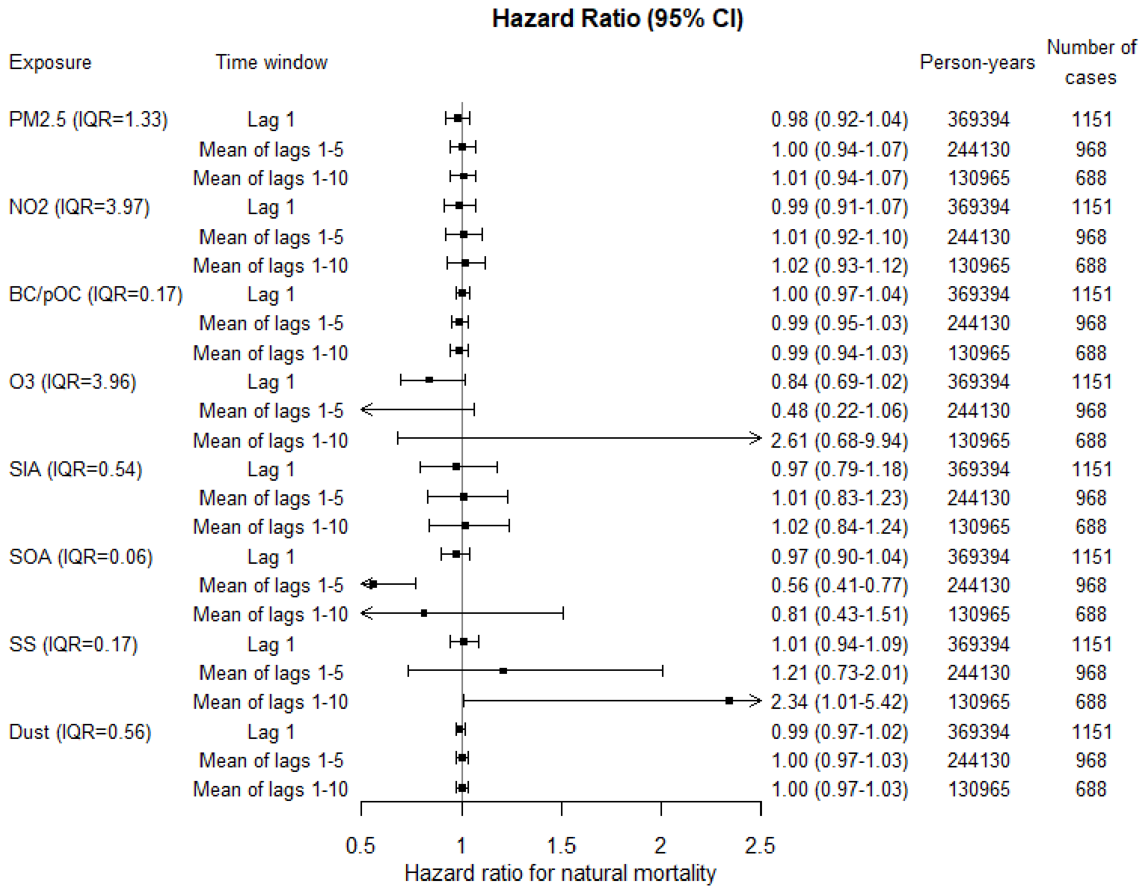

Figure S3a. Unadjusted hazard ratios for natural mortality per inter-quartile range (IQR), excluding individuals with missing information on covariates.

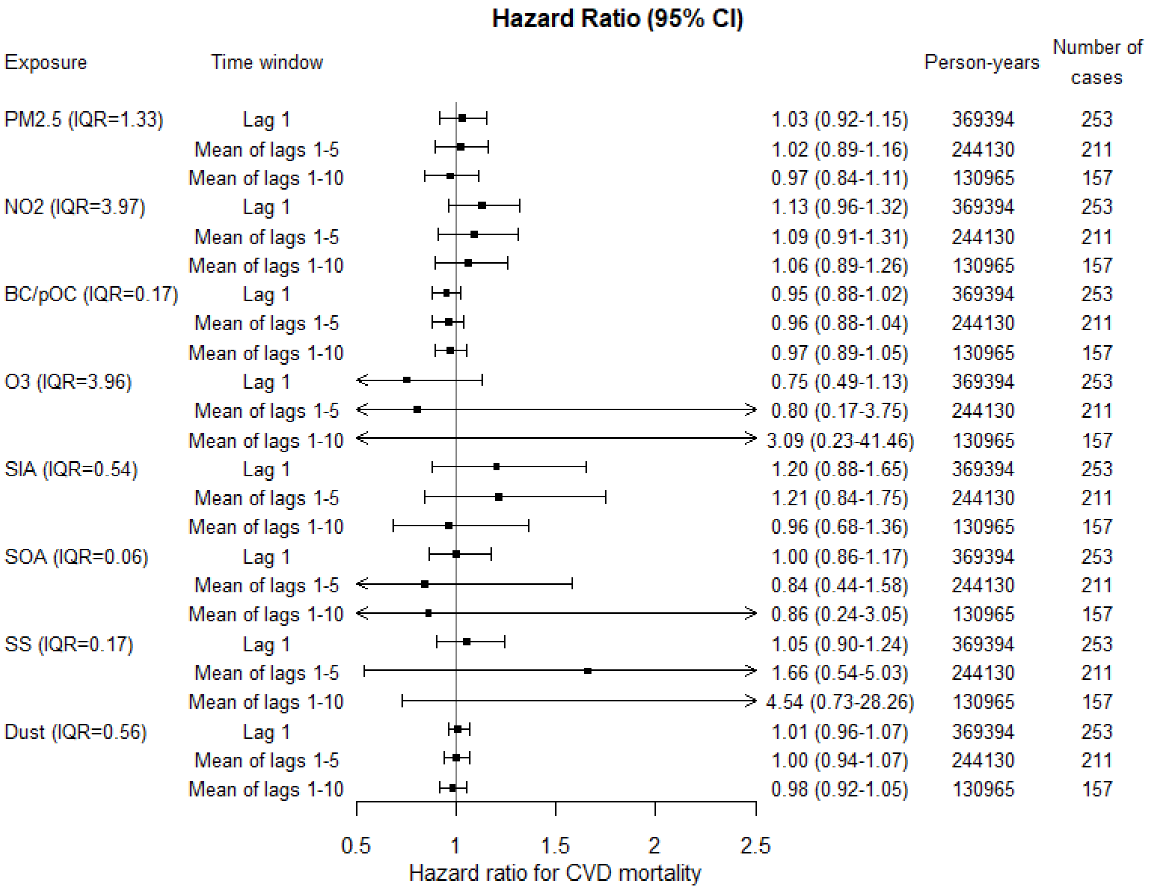

Figure S3b. Unadjusted hazard ratios for CVD mortality per inter-quartile range (IQR), excluding individuals with missing information on covariates.

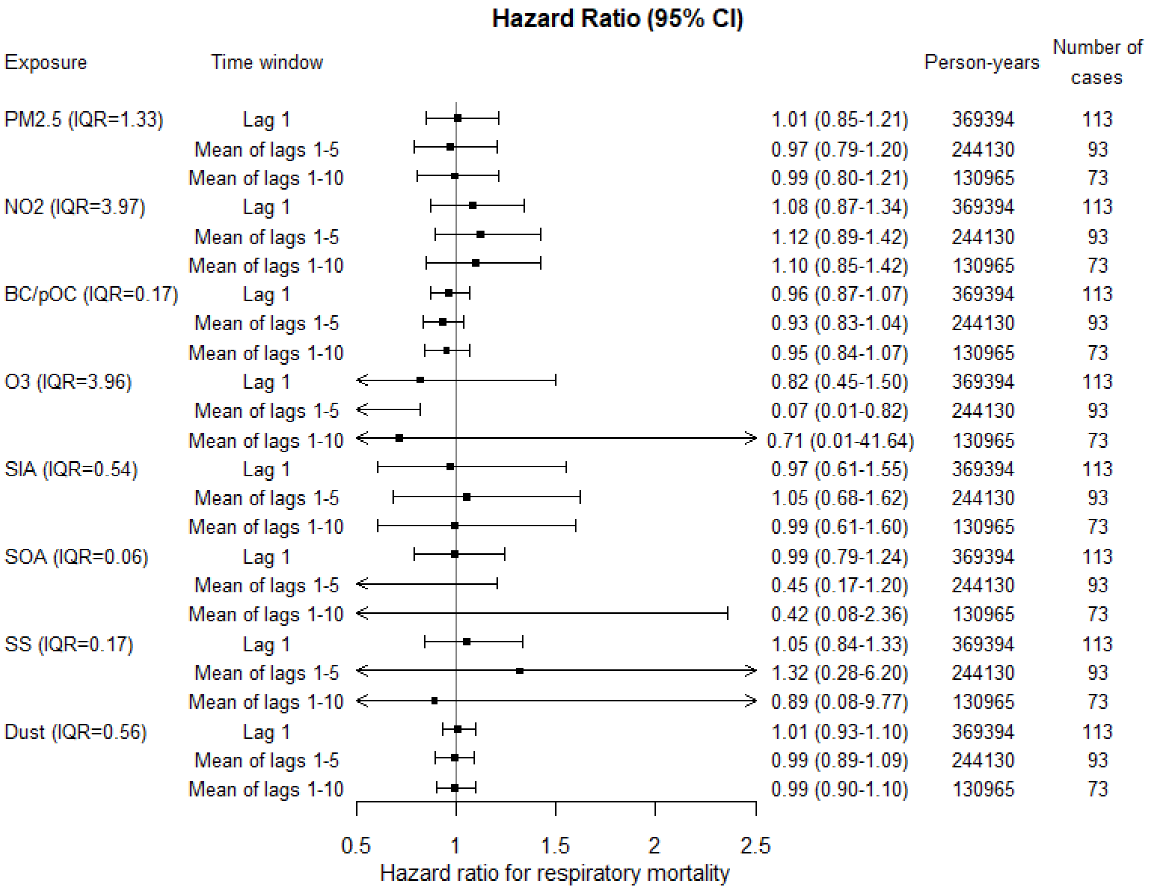

Figure S3c. Unadjusted hazard ratios for respiratory mortality per inter-quartile range (IQR), excluding individuals with missing information on covariates.

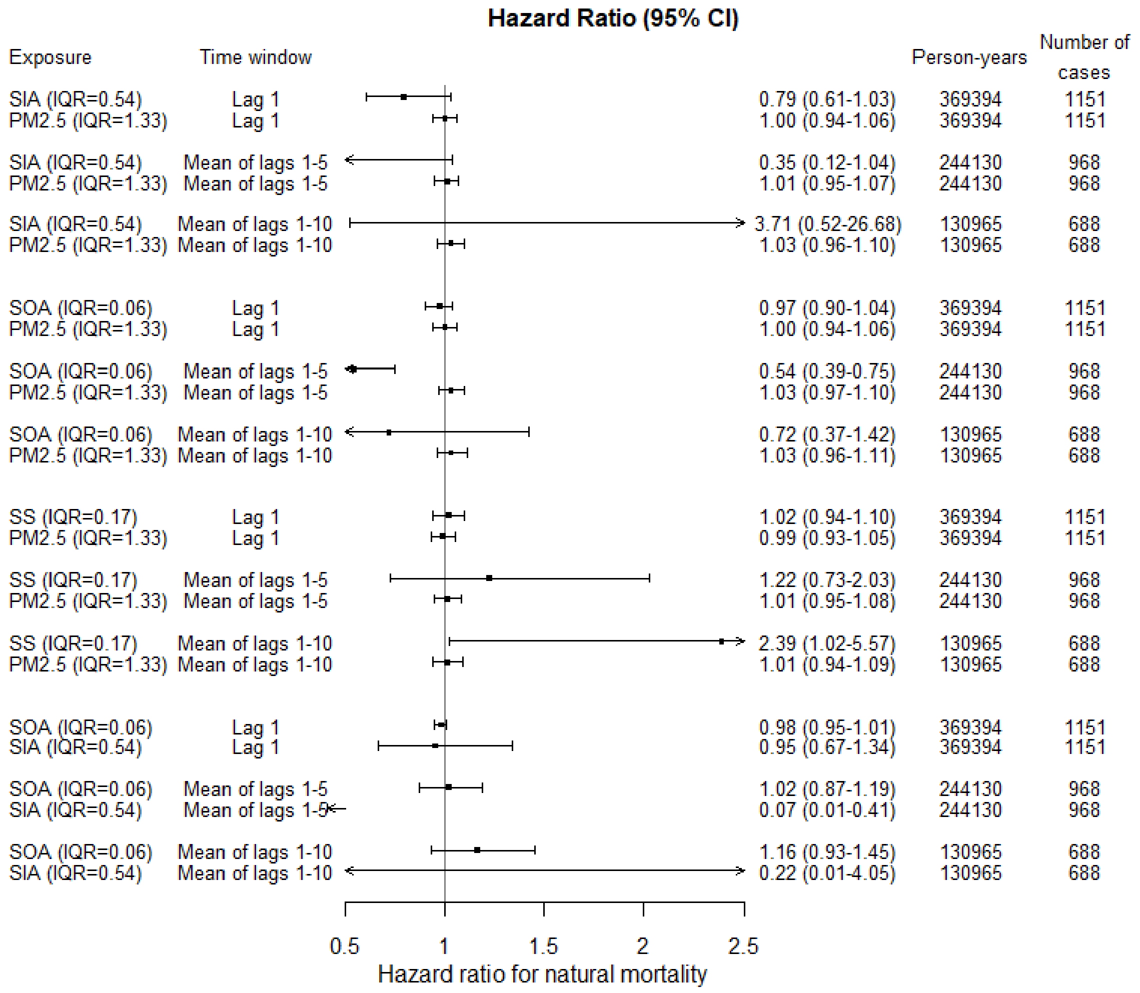

Figure S4a. Adjusted hazard ratios for natural mortality per inter-quartile range (IQR) in two-pollutant models; SIA, SOA and SS adjusted for total $PM_{2.5}$, and mutually adjusted estimates for SIA and SOA.

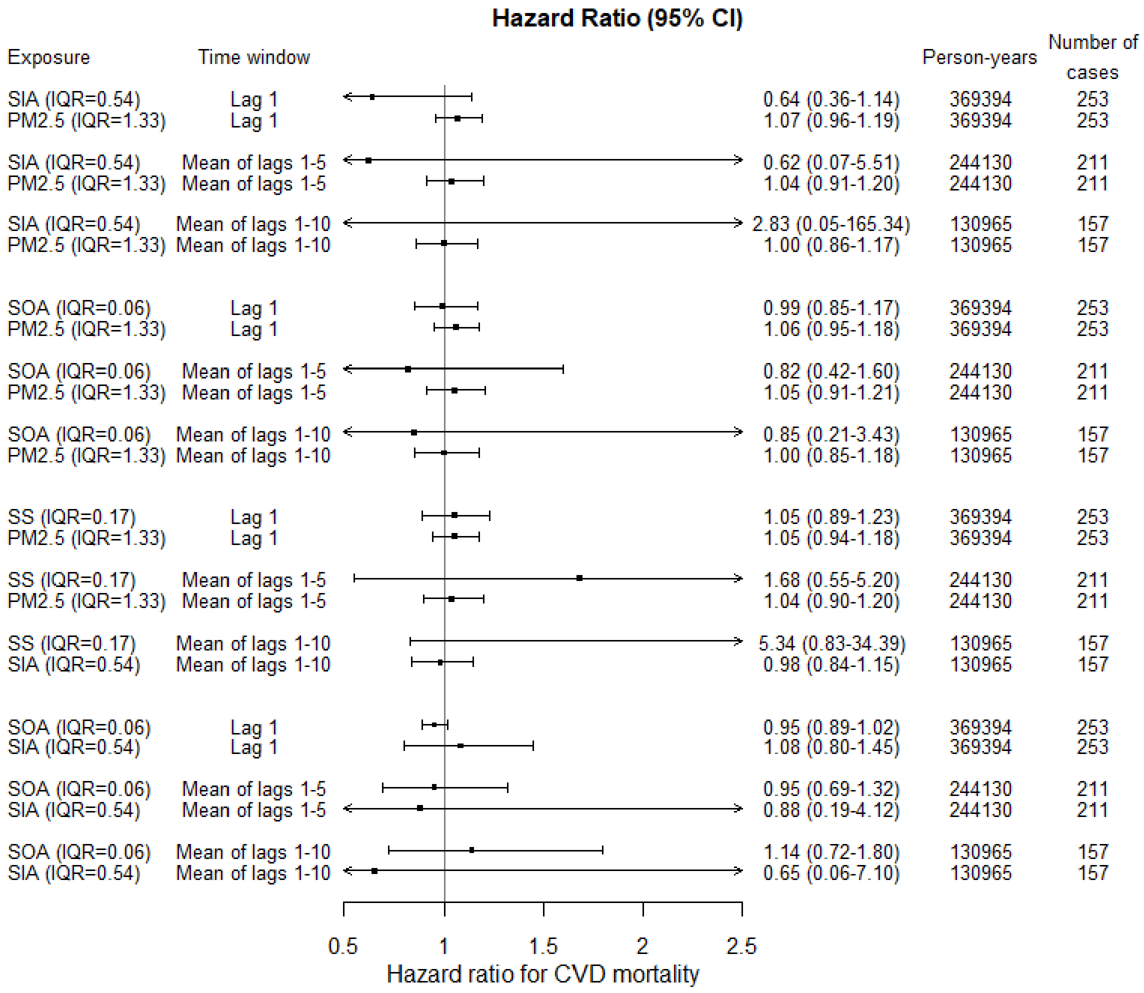

Figure S4b. Adjusted hazard ratios for CVD mortality per inter-quartile range (IQR) in two-pollutant models; SIA, SOA and SS adjusted for total $PM_{2.5}$, and mutually adjusted estimates for SIA and SOA.

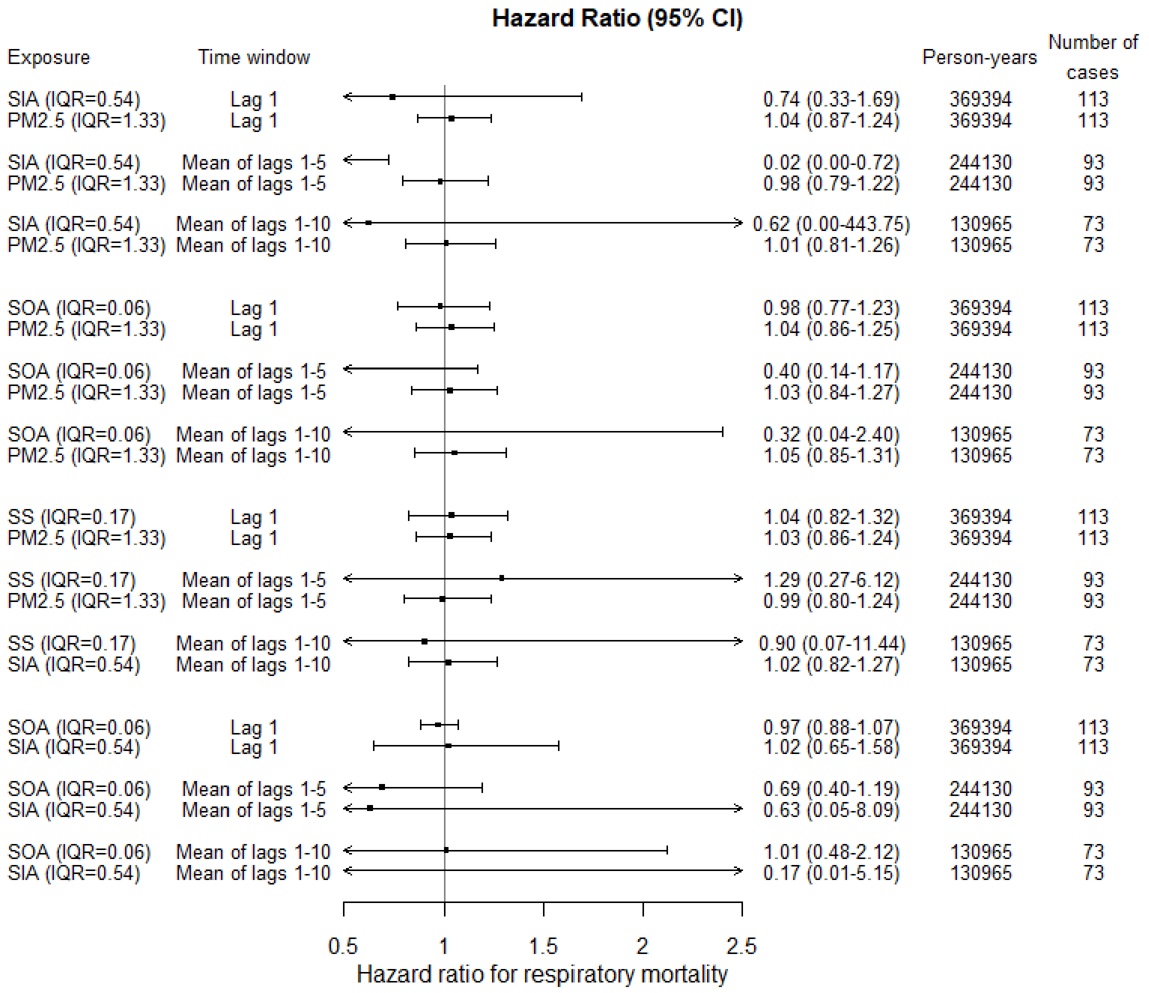

Figure S4c. Adjusted hazard ratios for respiratory mortality per inter-quartile range (IQR) in two-pollutant models; SIA, SOA and SS adjusted for total $PM_{2.5}$, and mutually adjusted estimates for SIA and SOA.
